# Qualitative Analysis of a Nonautonomous Delayed Stochastic Predator–Prey Model with Beddington–DeAngelis Functional Response

**DOI:** 10.3390/biology14081078

**Published:** 2025-08-18

**Authors:** Lili Jia, Changyou Wang

**Affiliations:** 1 Department of Basic Teaching, Dianchi College, Kunming 650228, China; lilijiadianchi@163.com; 2 School of Mathematical Sciences, Sichuan Normal University, Chengdu 610066, China; 3 College of Applied Mathematics, Chengdu University of Information Technology, Chengdu 610225, China

**Keywords:** delays, nonautonomous, Beddington–DeAngelis, uniform persistence, global attractivity

## Abstract

In this paper, we propose a novel stochastic multi-species predator–prey model that integrates time delays and the Beddington–DeAngelis functional response, marking a significant advancement in ecological modeling under environmental stochasticity. This model explicitly accounts for environmental fluctuations by perturbing intrinsic growth and death rates, offering a more realistic and nuanced portrayal of complex predator–prey interactions. Our primary objectives are to establish the existence and uniqueness of a global positive solution for any positive initial value, derive sufficient conditions for the uniform persistence (permanent coexistence) of all species, and investigate the global stability of the system’s solutions. By constructing sophisticated Lyapunov functions tailored to the model’s stochastic and delayed characteristics, we derive rigorous criteria that ensure the global stability of solutions, which constitutes a notable achievement considering the system’s inherent complexity. Additionally, we formulate sufficient conditions for uniform persistence, providing a theoretical foundation for understanding the long-term survival and stability of multi-species ecosystems under random environmental disturbances and historical effects. To validate our theoretical findings, we conduct extensive numerical simulations using the Milstein method. These simulations not only corroborate our analytical results but also elucidate the dynamic behaviors of the model, demonstrating system permanence within bounded densities, the convergence of solutions under varying initial conditions, and the extinction of the top predator when theoretical conditions are not met. This analysis highlights the necessity of the derived criteria for maintaining ecological balance. The analytical framework developed here lays a solid mathematical foundation for understanding the periodic and long-term behaviors of complex food chains under environmental stochasticity, with significant implications for the conservation and management of biological populations.

## Simple Summary

This study creates a new ecological model to investigate how multiple predator and prey species interact under unpredictable environmental changes. Unlike previous models, it incorporates realistic factors such as delayed species reactions and specific hunting patterns. The researchers aimed to demonstrate long-term survival of all species (permanence), confirm global stability of population dynamics, and verify these outcomes through simulations. Key findings reveal that populations stabilize within healthy ranges when specific ecological conditions are met. However, if these conditions deteriorate—for example, when top predators find there is insufficient prey—their populations may decline toward extinction. This model provides a mathematical framework for anticipating and managing ecosystems affected by environmental randomness, supporting efforts to conserve biodiversity and maintain ecological harmony. By clarifying how response delays and species interactions influence survival, the research aids sustainable strategies protecting wildlife in shifting climates. Understanding these dynamics helps policymakers design interventions to prevent imbalances, ensuring healthier ecosystems for future generations.

## 1. Introduction

Predator–prey interactions constitute a fundamental dynamic governing population regulation, community structure, and ecosystem stability [1,2]. While classical deterministic models like the Lotka–Volterra framework provide foundational insights, they often fail to capture the inherent complexities of natural systems. For example, in 1999, Conser et al. [3] demonstrated the rationality of incorporating ratio-dependent terms into predator–prey models by analyzing discrepancies in the foundational principles of predation dynamics. In 2013, Muhammadhaji et al. [4] discovered through their research that time delay significantly influences the persistence of predator–prey systems. More recently, in 2025, Roy et al. [5] introduced stochastic perturbations into the Lotka–Volterra framework, revealing the impact of environmental white noise and demonstrating through research that noise can induce changes in the stable states of the system. To bridge this gap, contemporary ecological modeling integrates the following three critical dimensions: time-delays, nonlinear functional responses, and environmental stochasticity. Incorporating these elements is essential for developing realistic predictions about species coexistence and ecosystem resilience [6,7].

Time-delays explicitly account for ubiquitous biological lags inherent in ecological processes [8]. These delays arise from gestation periods, resource regeneration times, predator handling and digestion of prey, or juvenile maturation before reproductive contribution. Neglecting these temporal components can lead to the significant misestimation of the stability thresholds and oscillatory behavior, as delays fundamentally alter system dynamics, potentially destabilizing equilibria or inducing complex cycles [9,10,11]. The functional response, describing the per capita predator consumption rate as prey density varies, is another vital component. The Beddington–DeAngelis (BD) functional response, characterized by terms like $\frac{a_{ij}x_{j}}{1+\beta_{i}x_{i}+\gamma_{j}x_{j}}$, offers a significant mechanistic advance over simpler Holling types [12,13]. It incorporates mutual interference among predators (via $\gamma_{j}x_{j}$) and potential interference from prey or other species (via $\beta_{i}x_{i}$), providing a more general and biologically grounded description of trophic interactions where crowding or defensive behaviors modulate consumption rates [14,15]. Crucially, populations persist in environments characterized by pervasive, unpredictable fluctuations. Deterministic models inherently fail to capture the profound influence of environmental stochasticity stemming from random climatic events, habitat disturbances, disease outbreaks, or anthropogenic impacts [16]. Stochastic differential equations (SDEs) provide the essential mathematical framework that can incorporate this intrinsic randomness [17]. By modeling key parameters (e.g., intrinsic growth/death rates) as stochastic processes, SDEs yield robust predictions about population persistence, extinction risk, and the distribution of population sizes under uncertainty [18,19]. The intricate interplay between noise intensity, time delays, and nonlinear functional responses can induce phenomena absent in deterministic models, such as noise-induced transitions or modified persistence–extinction boundaries [20,21,22,23].

There has been extensive research on ecological models, but they often focus on just one or two key elements. For instance, some models incorporate the BD functional response [24], while others explore stochastic models without delay [25]. There are also models that combine two factors, such as the BD functional response with stochastic noise [18] or stochastic noise with delay [20]. However, integrating all three factors—delay, BD functional response, and stochastic noise—into a multi-species framework remains a critical and challenging task in ecological modeling research. Understanding how these factors collectively govern population dynamics, coexistence (uniform persistence), and long-term convergence (global attractivity) is paramount for advancing ecological theory and informing practical applications in conservation and resource management.

This paper addresses this gap by proposing and rigorously analyzing a stochastic multi-species predator–prey model incorporating discrete time-delays and BD functional responses. Building upon deterministic foundations like the following model

(1) $$\left\{\begin{array}{l} {\dot{u}}_{1}(t)=u_{1}(t)\left[a_{1}(t)-b_{1}(t)u_{1}(t)-\frac{s_{1}(t)u_{2}(t)}{A_{1}(t)+u_{1}(t)+B_{1}(t)u_{2}(t)}\right],\\ {\dot{u}}_{2}(t)=u_{2}(t)\left[-a_{2}(t)-b_{2}(t)u_{2}(t)+\frac{s_{3}(t)u_{1}(t-\tau )}{A_{1}(t)+u_{1}(t-\tau )+B_{1}(t)u_{2}(t-\tau )}-\frac{s_{2}(t)u_{3}(t)}{A_{2}(t)+u_{2}(t)+B_{2}(t)u_{3}(t)}\right],\\ {\dot{u}}_{3}(t)=u_{3}(t)\left[-a_{3}(t)-b_{3}(t)u_{3}(t)+\frac{s_{4}(t)u_{2}(t-\tau )}{A_{2}(t)+u_{2}(t-\tau )+B_{2}(t)u_{3}(t-\tau )}\right], \end{array}\right.$$

studied by Xu et al. [26], we introduce stochastic perturbations reflecting environmental noise, leading to the following system

(2) $$\left\{\begin{array}{l} dx_{1}(t)=x_{1}(t)\left[r_{1}(t)-a_{11}(t)x_{1}(t)-\frac{a_{12}(t)x_{2}(t)}{1+\beta_{1}(t)x_{1}(t)+\gamma_{1}(t)x_{2}(t)}\right]dt+\sigma_{1}(t)x_{1}(t)dB_{1}(t),\\ \begin{array}{l} dx_{2}(t)=x_{2}(t)\left[-r_{2}(t)+\frac{a_{21}(t)x_{1}(t-\tau_{1})}{1+\beta_{1}(t)x_{1}(t-\tau_{1})+\gamma_{1}(t)x_{2}(t-\tau_{1})}-a_{22}(t)x_{2}(t)-\frac{a_{23}(t)x_{3}(t)}{1+\beta_{2}(t)x_{2}(t)+\gamma_{2}(t)x_{3}(t)}\right]dt\\ \;\;\;+\sigma_{2}(t)x_{2}(t)dB_{2}(t), \end{array}\\ dx_{3}(t)=x_{3}(t)\left[-r_{3}(t)+\frac{a_{32}(t)x_{2}(t-\tau_{2})}{1+\beta_{2}(t)x_{2}(t-\tau_{2})+\gamma_{2}(t)x_{3}(t-\tau_{2})}-a_{33}(t)x_{3}(t)\right]dt+\sigma_{3}(t)x_{3}(t)dB_{3}(t), \end{array}\right.$$

with the following boundary and initial conditions

(3) $$x_{i}(t)=\phi_{i}(t)>0,\;\;t\in [-\tau ,0],\;\;i=1,2,3,\tau =\max\left\{\tau_{1}\right.,\tau_{2}\},$$

where $x_{i}(t)(i=1,2,3)$, respectively, denote the density of the prey population, predator population, and top predator population at time t. $\sigma_{i}(t)\;dB_{i}(t)\;(i=1,2,3)$ are stochastic items, $B_{i}(t)$ is the one-dimensional standard Brownian motion defined on a complete probability space (Ω,F,P), and $\sigma_{i}(t)$ represents the intensity of the noise. Considering that the introduction of the stochastic term is to regulate the intrinsic growth rate of the biological population, and taking into account the biological significance of the parameters $B_{i}(t)$, we assume $-r_{i}(t)\leq B_{i}(t)\leq r_{i}(t),$ i=1,2,3. From Table 1, one can discern the biological implications associated with the other parameters in model (2). All coefficients in the model are continuous, bounded, and strictly positive functions on the interval [0,+∞).

In this paper, we will establish the fundamental properties of a stochastic delayed predator–prey model with BD functional response, first proving the existence and uniqueness of a global positive solution for any positive initial value to ensure mathematical well-posedness. Building on this foundation, we derive explicit sufficient algebraic conditions that guarantee the persistence of the biology system, ensuring all populations remain bounded away from extinction thresholds throughout their long-term evolutionary trajectories. To characterize asymptotic behavior, novel Lyapunov functionals are constructed to establish sufficient criteria for global attractivity, demonstrating the almost sure convergence of solutions to a unique equilibrium regardless of initial conditions. Finally, comprehensive numerical validations via the Milstein method will empirically verify all theoretical results on solution behavior, persistence thresholds, and attractivity dynamics.

Following the establishment of core analytical objectives, this paper is organized as follows: Section 2 rigorously proves the existence and uniqueness of a global positive solution using the existence and uniqueness theorem of the solution of the delayed stochastic differential equation and the continuous dependence of the solution on the initial value. Section 3 derives sufficient algebraic conditions for uniform persistence through comparative analysis of auxiliary stochastic systems and limit superior/inferior estimates, explicitly linking parameter bounds to coexistence thresholds. Section 4 constructs novel Lyapunov functionals incorporating delay compensation terms to establish global attractivity criteria, with stability analysis leveraging semi-martingale convergence theory. Section 5 implements the Milstein method for numerical simulations, validating theoretical thresholds and demonstrating noise-induced dynamical regimes. Finally, Section 6 synthesizes biological implications, limitations, and future research directions.

This work makes four significant advances as follows: (1) It introduces the first unified framework integrating multi-species interactions, discrete delays, Beddington–DeAngelis responses, and multiplicative noise in a single stochastic model, generalizing prior studies limited to subsets of these features. (2) The derived explicit persistence criteria (Theorem 2 establish quantitatively verifiable thresholds involving noise intensities $\left(\sigma_{i}\right)$, delay bounds $\left(\tau_{i}\right)$, and interaction coefficients $\left(\alpha_{ij},\beta_{i},\gamma_{j}\right)$, revealing how environmental stochasticity modulates coexistence windows. (3) The construction of delay-embedded Lyapunov functionals (Theorem 3) overcomes technical barriers in proving almost sure convergence for coupled nonlinear stochastic-delay systems, providing a generalizable method for attractivity analysis. (4) Numerical experiments demonstrate noise-driven regime shifts, including stochastic resonance near persistence boundaries and delay-induced oscillation synchronization phenomena inaccessible to deterministic approximations. Collectively, these contributions bridge theoretical ecology and stochastic analysis, offering testable predictions for complex ecosystems under environmental volatility.

## 2. Existence and Uniqueness of the Global Positive Solution of Model (2)

Fix an arbitrary τ>0 and a positive integer d and consider the following random functional differential equations

(4) $$\left\{\begin{array}{l} {\dot{x}}_{i}(t)=f_{i}({ƛ}_{t}\omega ,x_{t}),\;i=1,2,\ldots ,d,\;t\geq 0,\\ x(\theta )=\phi (\theta )\in C,\;\theta \in [-\tau ,0], \end{array}\right.$$

and

(5) $$\left\{\begin{array}{l} {\dot{y}}_{i}(t)=F_{i}({ƛ}_{t}\omega ,y_{t}),\;i=1,2,\ldots ,d,\;t\geq 0,\\ y(\theta )=\psi (\theta )\in C,\;\theta \in [-\tau ,0], \end{array}\right.$$

where ${ƛ}_{t}\omega :\Omega \to \Omega ,\;t\in R$ is a metric dynamical system, $x(t)={\left(x_{1}(t),x_{2}(t),\ldots ,x_{d}(t)\right)}^{T}$, $y(t)={\left(y_{1}(t),y_{2}(t),\ldots ,y_{d}(t)\right)}^{T}$, $x_{t}(\theta )=x(t+\theta )$, $y_{t}(\theta )=y(t+\theta )$, and C is the Banach space of all the continuous functions $\gamma :J\to R^{d}$ with the sup-norm $\left\|\gamma \right\|=\sup_{s\in J}\left|\gamma (s)\right|$, where $\left|•\right|$ denotes the Euclidean norm of a vector. $f=(f_{1},f_{2},\ldots ,f_{d})$ and $F=(F_{1},F_{2},\ldots ,F_{d})$ are measurable vector functions from $\Omega \times R^{d}$ to $R^{d}$.

**Lemma 1**  ([27], Proposition 2.4)**.** *Suppose that f satisfies the locally bounded and local Lipschitz conditions. Then for each ω∈Ω and ϕ∈C, model (4) has a unique non-continuable solution x(t)=x(t,ω,ϕ) on $\left[0,t_{M}(\omega ,\phi )\right)$, and if* $t_{M}=t_{M}(\omega ,\varphi )<+\infty$*, then $\underset{t\to t_{M}}{\lim}\left\|x(t)\right\|=+\infty$; for each* ω∈Ω*, the solution* x(t):(t,ϕ)→x(t,ω,ϕ) *is continuous on* $\left\{(t,\phi ):\;\phi \in C,\;0\leq t<t_{M}\right\}$.

**Theorem 1.**  *For any given initial value* $(\phi_{1}(t),\phi_{2}(t),\phi_{3}(t))\in C([-\tau ,0],R_{+}^{3}),$ *models (2)–(3) have a unique, positive and global solution.*

**Proof.**  Due to the continuity of the functions on the right-hand side of model (2) and its satisfaction of the local Lipschitz condition, models (2)–(3) possess a unique local solution, denoted as $x_{1}(t),\;x_{2}(t)$ and $x_{3}(t)$, on a small interval [0, T) according to the existence and uniqueness theorem for solutions of random functional differential equations (See Lemma 1). Next, we prove that for any positive initial values, this local solution remains positive and can be extended to the entire positive time axis.

According to the first equation of model (2), when $x_{1}(t)>0,x_{2}(t)>0$ and $x_{3}(t)>0$, the sign of the right-hand side of the equation depends on

$$\Delta_{1}(t)≜r_{1}(t)-a_{11}(t)x_{1}(t)-\frac{a_{12}(t)x_{2}(t)}{1+\beta_{1}(t)x_{1}(t)+\gamma_{1}(t)x_{2}(t)}.$$

If the initial value results in $\Delta_{1}(t)>0$, then the rate of change of $x_{1}(t)$ is positive, and since the initial value $\phi_{1}(t)>0$ is positive, $x_{1}(t)$ will remain positive. If the initial values result in $\Delta_{1}(t)<0$, then the rate of change of $x_{1}(t)$ is negative, and due to the positive initial value, the prey population $x_{1}(t)$ will decrease. This is based on the interaction mechanisms among populations in ecosystems and the continuity of population dynamics. Subsequently, the predator population $x_{2}(t)$ will also decrease due to insufficient food. Since $r_{1}(t)>0$, as the populations of $x_{1}(t)$ and $x_{2}(t)$ decrease, eventually $\Delta_{1}(t)$ will become positive, causing the population of $x_{1}(t)$ to increase before reaching zero. In summary, regardless of whether the initial values make $\Delta_{1}(t)>0$ or $\Delta_{1}(t)<0$, $x_{1}(t)$ remains positive.

Similarly, according to the second equation of model (2), when $x_{1}(t)>0,x_{2}(t)>0$ and $x_{3}(t)>0$, the sign of the right-hand side of the equation depends on

$$\Delta_{2}(t)≜-r_{2}(t)+\frac{a_{21}(t)x_{1}(t-\tau_{1})}{1+\beta_{1}(t)x_{1}(t-\tau_{1})+\gamma_{1}(t)x_{2}(t-\tau_{1})}-a_{22}(t)x_{2}(t)-\frac{a_{23}(t)x_{3}(t)}{1+\beta_{2}(t)x_{2}(t)+\gamma_{2}(t)x_{3}(t)}.$$

If the initial values result in $\Delta_{2}(t)>0$, then the rate of change of $x_{2}(t)$ is positive, and since the initial value $\phi_{2}(t)>0$ is positive, $x_{2}(t)$ will remain positive. If the initial values result in $\Delta_{2}(t)<0$, then the rate of change of $x_{2}(t)$ is negative, and due to the positive initial value, the predator population $x_{2}(t)$ will continuously decrease. This is based on the interaction mechanisms among populations in ecosystems and the continuity of population dynamics. Simultaneously, the prey population $x_{1}(t)$ will increase due to the reduction in predator population and the top predator population $x_{3}(t)$ will decrease due to the reduction in predator population. As $x_{2}(t)$ and $x_{3}(t)$ decrease and $x_{1}(t)$ increases, eventually $\Delta_{2}(t)>0$ will become positive (since $a_{21}(t)>0$), causing the population of $x_{2}(t)$ to increase before decreasing to zero. In summary, regardless of whether the initial values make $\Delta_{2}(t)>0$ or $\Delta_{2}(t)<0$, $x_{2}(t)$ remains positive.

Furthermore, according to the third equation of model (2), when $x_{1}(t)>0,x_{2}(t)>0$, and $x_{3}(t)>0$, the sign of the right-hand side of the equation depends on

$$\Delta_{3}(t)≜-r_{3}(t)+\frac{a_{32}(t)x_{2}(t-\tau_{2})}{1+\beta_{2}(t)x_{2}(t-\tau_{2})+\gamma_{2}(t)x_{3}(t-\tau_{2})}-a_{33}(t)x_{3}(t).$$

If the initial values result in $\Delta_{3}(t)>0$, then the rate of change of $x_{3}(t)$ is positive, and since the initial value $\phi_{3}(t)>0$ is positive, $x_{3}(t)$ will remain positive. If the initial values result in $\Delta_{3}(t)<0$, then the rate of change of $x_{3}(t)$ is negative, and due to the positive initial value, the top predator population $x_{3}(t)$ will continuously decrease. This is based on the interaction mechanisms among populations in ecosystems and the continuity of population dynamics. Simultaneously, the predator population $x_{2}(t)$ will increase due to the reduction in the top predator population. As $x_{3}(t)$ decreases and $x_{2}(t)$ increases, eventually $\Delta_{2}(t)>0$ will become positive (since $a_{32}(t)>0$), causing the population of $x_{3}(t)$ to increase before decreasing to zero. In summary, regardless of whether the initial values make $\Delta_{3}(t)>0$ or $\Delta_{3}(t)<0$, $x_{3}(t)$ remains positive.

Finally, since the local solution is unique and positive, we can utilize the continuous dependence theorem for solutions of random functional differential equations (See Lemma 1) to extend the local solution to the entire positive time axis while maintaining its positivity. Therefore, given the initial conditions $\phi_{1}(t)>0,\phi_{2}(t)>0$ and $\phi_{3}(t)>0$, the solutions of the predator–prey models (2)–(3) remain positive on the entire positive time axis. ☐

## 3. Uniform Permanence of Model (2)

For convenience of description, for any continuous and bounded function g(t) on $\left[t_{0},+\infty \right)$, we introduce the symbols

$$g^{m}=\sup\{g(t)|t_{0}\leq t<+\infty \},g^{l}=\inf\{g(t)|t_{0}\leq t<+\infty \}.$$

**Definition 1.**  *Assuming that for any solution* $\Phi (t)=(x_{1}(t),x_{2}(t),x_{3}(t))$ *of model (2) that satisfies the initial condition (3) there exists positive number $m_{i},M_{i}(i=1,2,3)$ and T, such that*

$$m_{i}\leq x_{i}(t)\leq M_{i},\;(i=1,2,3),\;t\geq T,$$

*then, model (2) is said to be the uniform permanence.*

**Lemma 2**  ([28], Theorem 2.2)**.** *Consider the following equation*

dN(t)=N(t)[(a(t)−b(t)N(t))dt+α(t)dB(t)],

*where* a(t),b(t)*, and* α(t) *are bounded continuous functions defined on* [0,+∞)*. If* a(t)>0 *and* b(t)>0, *then there exists a unique continuous positive solution* N(t) *for any initial value* $N(0)=N_{0}>0$*, which is global and represented by*

$$N(t)=\frac{\exp\{\int_{0}^{t}\left[a(s)-0.5\alpha^{2}(s)\right]ds+\alpha (s)dB(s)\}}{1/N_{0}+\int_{0}^{t}b(s)\exp\{\int_{0}^{s}\left[a(\theta )-0.5\alpha^{2}(\theta )\right]d\theta +\alpha (\theta )dB(\theta )\}ds},\;t\geq 0.$$

**Lemma 3**  ([27], Theorem 3.1)**.** *Suppose that f and F satisfy the locally bounded and local Lipschitz conditions, the inequality $f_{i}(\omega ,\;\phi )\leq F_{i}(\omega ,\;\psi )$ is fulfilled whenever* ϕ≤ψ*, $\phi_{i}(0)=\psi_{i}(0)$ holds for* i *and all* ω∈Ω*, and ϕ, ψ∈C. If for a given* $\omega_{0}\in \Omega$*, the solutions* $x(t,\omega_{0},\phi )$ *and $y(t,\omega_{0},\psi )$ of random functional differential Equations (4) and (5) are defined on [0,T), then $x(t,\omega_{0},\phi )\leq y(t,\omega_{0},\psi )$ for all* t∈[0,T)*. Furthermore, if all the solutions are global, then* $x(t,\omega_{0},\phi )\leq y(t,\omega_{0},\psi )$ *for all* t≥0 *and ω∈Ω*.

**Theorem 2.**  *Assuming that model (2) satisfies initial condition (3), the following conditions are met:*

$$\begin{array}{l} (H_{1})\;r_{1}^{m}>0.5{\left(\sigma_{1}^{l}\right)}^{2},\;(H_{2})\;a_{21}^{m}/\beta_{1}^{l}>r_{2}^{l}+0.5{\left(\sigma_{2}^{l}\right)}^{2},\;(H_{3})\;a_{32}^{m}/\beta_{2}^{l}>r_{3}^{l}+0.5{\left(\sigma_{3}^{l}\right)}^{2},\\ (H_{4})\;r_{1}^{l}>a_{12}^{m}/\gamma_{1}^{l}+0.5{\left(\sigma_{1}^{m}\right)}^{2},\;(H_{5})a_{21}^{l}m_{1}/(1+\beta_{1}^{m}M_{1}+\gamma_{1}^{m}M_{2})>r_{2}^{m}+a_{23}^{m}/\gamma_{2}^{l}+0.5{\left(\sigma_{2}^{m}\right)}^{2},\\ (H_{6})\;a_{32}^{l}m_{2}/(1+\beta_{2}^{m}M_{2}+\gamma_{2}^{m}M_{3})>r_{3}^{m}+0.5{\left(\sigma_{3}^{m}\right)}^{2}. \end{array}$$

Then model (2) is the uniform persistence, where

$$\begin{array}{l} M_{1}=\frac{r_{1}^{m}-0.5{\left(\sigma_{1}^{l}\right)}^{2}}{a_{11}^{l}},\;M_{2}=\frac{-r_{2}^{l}+a_{21}^{m}/\beta_{1}^{l}-0.5{\left(\sigma_{2}^{l}\right)}^{2}}{a_{22}^{l}},\\ M_{3}=\frac{-r_{3}^{l}+a_{32}^{m}/\beta_{2}^{l}-0.5{\left(\sigma_{3}^{l}\right)}^{2}}{a_{33}^{l}},\;m_{1}=\frac{r_{1}^{l}-a_{12}^{m}/\gamma_{1}^{l}-0.5{\left(\sigma_{1}^{m}\right)}^{2}}{a_{11}^{m}},\\ m_{2}=\frac{-r_{2}^{m}+a_{21}^{l}m_{1}/(1+\beta_{1}^{m}M_{1}+\gamma_{1}^{m}M_{2})-a_{23}^{m}/\gamma_{2}^{l}-0.5{\left(\sigma_{2}^{m}\right)}^{2}}{a_{22}^{m}},\\ m_{3}=\frac{-r_{3}^{m}+a_{32}^{l}m_{2}/(1+\beta_{2}^{m}M_{2}+\gamma_{2}^{m}M_{3})-0.5{\left(\sigma_{3}^{l}\right)}^{2}}{a_{33}^{m}}. \end{array}$$

**Proof.**  For the first equation of model (2), there is

$$\begin{array}{l} dx_{1}(t)=x_{1}(t)\left[r_{1}(t)-a_{11}(t)x_{1}(t)-\frac{a_{12}(t)x_{2}(t)}{1+\beta_{1}(t)x_{1}(t)+\gamma_{1}(t)x_{2}(t)}\right]dt+\sigma_{1}(t)x_{1}(t)dB_{1}(t)\\ \;\;\;\leq x_{1}(t)[r_{1}(t)-a_{11}(t)x_{1}(t)]dt+\sigma_{1}(t)x_{1}(t)dB_{1}(t)\\ \;\;\;\leq x_{1}(t)[r_{1}^{m}-a_{11}^{l}x_{1}(t)]dt+\sigma_{1}^{m}x_{1}(t)dB_{1}(t). \end{array}$$

If condition $\left(H_{1}\right)$ is satisfied, according to Lemmas 2 and 3, we have

(6) $$\begin{array}{ll} \underset{t\to \infty }{\lim\sup}\;x_{1}(t) & \leq \underset{t\to \infty }{\lim}\frac{\exp\{\int_{0}^{t}\left[r_{1}(s)-0.5\sigma_{1}^{2}(s)\right]ds+\sigma_{1}(s)dB_{1}(s)\}}{1/\phi_{1}(t_{0})+\int_{0}^{t}a_{11}(s)\exp\{\int_{0}^{s}\left[r_{1}(\theta )-0.5\sigma_{1}^{2}(\theta )\right]d\theta +\sigma_{1}(\theta )dB_{1}(\theta )\}ds}\\ & \leq \underset{t\to \infty }{\lim}\frac{\exp\{[r_{1}^{m}-0.5{\left(\sigma_{1}^{l}\right)}^{2}]t+\sigma_{1}^{m}B_{1}^{m}\}}{1/\phi_{1}(t_{0})+a_{11}^{l}\int_{0}^{t}\exp\{[r_{1}^{m}-0.5{\left(\sigma_{1}^{l}\right)}^{2}]s+\sigma_{1}^{m}B_{1}^{m}\}ds}\\ & =\underset{t\to \infty }{\lim}\frac{\left[r_{1}^{m}-0.5{\left(\sigma_{1}^{l}\right)}^{2}]\phi_{1}(t_{0}\right)}{\exp\{-[r_{1}^{m}-0.5{\left(\sigma_{1}^{l}\right)}^{2}]t\}\{\exp(-\sigma_{1}^{m}B_{1}^{m})[r_{1}^{m}-0.5{\left(\sigma_{1}^{l}\right)}^{2}]-\phi_{1}(t_{0})a_{11}^{l}\}+\phi_{1}(t_{0})a_{11}^{l}}\\ & =\frac{r_{1}^{m}-0.5{\left(\sigma_{1}^{l}\right)}^{2}}{a_{11}^{l}}=M_{1}. \end{array}$$

From the second and third equations of model (2), there are

$$\begin{array}{ll} dx_{2}(t) & \leq x_{2}(t)\left[-r_{2}(t)+\frac{a_{21}(t)x_{1}(t-\tau_{1})}{1+\beta_{1}(t)x_{1}(t-\tau_{1})+\gamma_{1}(t)x_{2}(t-\tau_{1})}-a_{22}(t)x_{2}(t)\right]dt+\sigma_{2}(t)x_{2}(t)dB_{2}(t),\\ & \leq x_{2}(t)[-r_{2}(t)+\frac{a_{21}(t)}{\beta_{1}(t)}-a_{22}(t)x_{2}(t)]dt+\sigma_{2}(t)x_{2}(t)dB_{2}(t)\\ & \leq x_{2}(t)[-r_{2}^{l}+\frac{a_{21}^{m}}{\beta_{1}^{l}}-a_{22}^{l}x_{2}(t)]dt+\sigma_{2}^{m}x_{2}(t)dB_{2}(t), \end{array}$$

and

$$\begin{array}{ll} dx_{3}(t) & =x_{3}(t)\left[-r_{3}(t)+\frac{a_{32}(t)x_{2}(t-\tau_{2})}{1+\beta_{2}(t)x_{2}(t-\tau_{2})+\gamma_{2}(t)x_{3}(t-\tau_{2})}-a_{33}(t)x_{3}(t)\right]dt+\sigma_{3}(t)x_{3}(t)dB_{3}(t)\\ & \leq x_{3}(t)[-r_{3}(t)+\frac{a_{32}(t)}{\beta_{2}(t)}-a_{33}(t)x_{3}(t)]dt+\sigma_{3}(t)x_{3}(t)dB_{3}(t)\\ & \leq x_{3}(t)[-r_{3}^{l}+\frac{a_{32}^{m}}{\beta_{2}^{l}}-a_{33}^{l}(t)x_{3}(t)]dt+\sigma_{3}^{m}x_{3}(t)dB_{3}(t). \end{array}$$

Similarly, if conditions $\left(H_{2}),(H_{3}\right)$ are satisfied, according to Lemmas 2 and 3, we have

(7) $$\begin{array}{l} \underset{t\to \infty }{\lim\sup}\;x_{2}(t)\leq \underset{t\to \infty }{\lim}\frac{\exp\{[-r_{2}^{l}+a_{21}^{m}/\beta_{1}^{l}-0.5{\left(\sigma_{2}^{l}\right)}^{2}]t+\sigma_{2}^{m}B_{2}^{m}\}}{1/\phi_{2}(t_{0})+a_{22}^{l}\int_{0}^{t}\exp\{[-r_{2}^{l}+a_{21}^{m}/\beta_{1}^{l}-0.5{\left(\sigma_{2}^{l}\right)}^{2}]s+\sigma_{2}^{m}B_{2}^{m}\}ds}\\ \;\;\;\;\;=\frac{-r_{2}^{l}+a_{21}^{m}/\beta_{1}^{l}-0.5{\left(\sigma_{2}^{l}\right)}^{2}}{a_{22}^{l}}=M_{2}, \end{array}$$

and

(8) $$\begin{array}{l} \underset{t\to \infty }{\lim\sup}x_{3}(t)\leq \underset{t\to \infty }{\lim}\frac{\exp\{[-r_{3}^{l}+a_{32}^{m}/\beta_{2}^{l}-0.5{\left(\sigma_{3}^{l}\right)}^{2}]t+\sigma_{3}^{m}B_{3}^{m}\}}{1/\phi_{3}(t_{0})+a_{33}^{l}\int_{0}^{t}\exp\{[-r_{3}^{l}+a_{32}^{m}/\beta_{2}^{l}-0.5{\left(\sigma_{3}^{l}\right)}^{2}]s+\sigma_{3}^{m}B_{3}^{m}\}ds}\\ \;\;\;\;\;=\frac{-r_{3}^{l}+a_{32}^{m}/\beta_{2}^{l}-0.5{\left(\sigma_{3}^{l}\right)}^{2}}{a_{33}^{l}}=M_{3}. \end{array}$$

On the other hand, from the first equation of model (2), it holds that

$$\begin{array}{l} dx_{1}(t)=x_{1}(t)\left[r_{1}(t)-a_{11}(t)x_{1}(t)-\frac{a_{12}(t)x_{2}(t)}{1+\beta_{1}(t)x_{1}(t)+\gamma_{1}(t)x_{2}(t)}\right]dt+\sigma_{1}(t)x_{1}(t)dB_{1}(t),\\ \;\;\;\geq x_{1}(t)[r_{1}(t)-a_{11}(t)x_{1}(t)-\frac{a_{12}(t)}{\gamma_{1}(t)}]dt+\sigma_{1}(t)x_{1}(t)dB_{1}(t)\\ \;\;\;\geq x_{1}(t)[r_{1}^{l}-\frac{a_{12}^{m}}{\gamma_{1}^{l}}-a_{11}^{m}x_{1}(t)]dt+\sigma_{1}^{l}x_{1}(t)dB_{1}(t). \end{array}$$

If condition $\left(H_{4}\right)$ is satisfied, according to Lemmas 2 and 3, we have

(9) $$\begin{array}{l} \;\;\underset{t\to \infty }{\lim\inf}\;x_{1}(t)\\ \geq \underset{t\to \infty }{\lim}\frac{\exp\{\int_{0}^{t}\left[r_{1}(s)-a_{12}(s)/\gamma_{1}(s)-0.5\sigma_{1}^{2}(s)\right]ds+\sigma_{1}(s)dB_{1}(s)\}}{1/\phi_{1}(t_{0})+\int_{0}^{t}a_{11}(s)\exp\{\int_{0}^{s}\left[r_{1}(\theta )-a_{12}(\theta )/\gamma_{1}(\theta )-0.5\sigma_{1}^{2}(\theta )\right]d\theta +\sigma_{1}(\theta )dB_{1}(\theta )\}ds}\\ \geq \underset{t\to \infty }{\lim}\frac{\exp\{[r_{1}^{l}-a_{12}^{m}/\gamma_{1}^{l}-0.5{\left(\sigma_{1}^{m}\right)}^{2}]t+\sigma_{1}^{l}B_{1}^{l}\}}{1/\phi_{1}(t_{0})+a_{11}^{m}\int_{0}^{t}\exp\{[r_{1}^{l}-a_{12}^{m}/\gamma_{1}^{l}-0.5{\left(\sigma_{1}^{m}\right)}^{2}]s+\sigma_{1}^{l}B_{1}^{l}\}ds}\\ =\underset{t\to \infty }{\lim}\frac{\left[r_{1}^{l}-a_{12}^{m}/\gamma_{1}^{l}-0.5{\left(\sigma_{1}^{m}\right)}^{2}]\phi_{1}(t_{0}\right)}{\exp\{-[r_{1}^{l}-a_{12}^{m}/\gamma_{1}^{l}-0.5{\left(\sigma_{1}^{m}\right)}^{2}]t\}\{\exp(-\sigma_{1}^{l}B_{1}^{l})[r_{1}^{l}-a_{12}^{m}/\gamma_{1}^{l}-0.5{\left(\sigma_{1}^{m}\right)}^{2}]-\phi_{1}(t_{0})a_{11}^{m}\}+\phi_{1}(t_{0})a_{11}^{m}}\\ =\frac{r_{1}^{l}-a_{12}^{m}/\gamma_{1}^{l}-0.5{\left(\sigma_{1}^{m}\right)}^{2}}{a_{11}^{m}}=m_{1}. \end{array}$$

According to the second and third equations of model (2), we have

$$\begin{array}{l} dx_{2}(t)\;\geq x_{2}(t)\left[-r_{2}(t)+\frac{a_{21}(t)x_{1}(t-\tau_{1})}{1+\beta_{1}(t)x_{1}(t-\tau_{1})+\gamma_{1}(t)x_{2}(t-\tau_{1})}-a_{22}(t)x_{2}(t)-\frac{a_{23}(t)}{\gamma_{2}(t)}\right]dt\\ \;\;\;\;\;+\sigma_{2}(t)x_{2}(t)dB_{2}(t)\\ \;\;\;\;\geq x_{2}(t)\left[-r_{2}^{m}+\frac{a_{21}^{l}m_{1}}{1+\beta_{1}^{m}M_{1}+\gamma_{1}^{m}M_{2}}-\frac{a_{23}^{m}}{\gamma_{2}^{l}}-a_{22}^{m}x_{2}(t)\right]dt+\sigma_{2}^{l}x_{2}(t)dB_{2}(t), \end{array}$$

and

$$\begin{array}{l} dx_{3}(t)\geq x_{3}(t)\left[-r_{3}(t)+\frac{a_{32}(t)x_{2}(t-\tau_{2})}{1+\beta_{2}(t)x_{2}(t-\tau_{2})+\gamma_{2}(t)x_{3}(t-\tau_{2})}-a_{33}(t)x_{3}(t)\right]dt\\ \;\;\;\;+\sigma_{3}(t)x_{3}(t)dB_{3}(t)\\ \;\;\;\geq x_{3}(t)\left[-r_{3}^{m}+\frac{a_{32}^{l}m_{2}}{1+\beta_{2}^{m}M_{2}+\gamma_{2}^{m}M_{3}}-a_{33}^{m}x_{3}(t)\right]dt+\sigma_{3}^{l}x_{3}(t)dB_{3}(t). \end{array}$$

If condition $\left(H_{5}),(H_{6}\right)$ are satisfied, according to Lemmas 2 and 3, we have

(10) $$\begin{array}{l} \underset{t\to \infty }{\lim\inf}\;x_{2}(t)\\ \geq \underset{t\to \infty }{\lim}\frac{\exp\{[-r_{2}^{m}+a_{21}^{l}m_{1}/(1+\beta_{1}^{m}M_{1}+\gamma_{1}^{m}M_{2})-a_{23}^{m}/\gamma_{2}^{l}-0.5{\left(\sigma_{2}^{m}\right)}^{2}]t+\sigma_{2}^{l}B_{2}^{l}\}}{1/\phi_{2}(t_{0})+a_{22}^{m}\int_{0}^{t}\exp\{[-r_{2}^{m}+a_{21}^{l}m_{1}/(1+\beta_{1}^{m}M_{1}+\gamma_{1}^{m}M_{2})-a_{23}^{m}/\gamma_{2}^{l}-0.5{\left(\sigma_{2}^{m}\right)}^{2}]s+\sigma_{2}^{l}B_{2}^{l}\}ds}\\ \;=\frac{-r_{2}^{m}+a_{21}^{l}m_{1}/(1+\beta_{1}^{m}M_{1}+\gamma_{1}^{m}M_{2})-a_{23}^{m}/\gamma_{2}^{l}-0.5{\left(\sigma_{2}^{m}\right)}^{2}}{a_{22}^{m}}=m_{2}, \end{array}$$

and

(11) $$\begin{array}{l} \underset{t\to \infty }{\lim\inf}\;x_{3}(t)\geq \underset{t\to \infty }{\lim}\frac{\exp\{[-r_{3}^{m}+a_{32}^{l}m_{2}/(1+\beta_{2}^{m}M_{2}+\gamma_{2}^{m}M_{3})-0.5{\left(\sigma_{3}^{m}\right)}^{2}]t+\sigma_{3}^{l}B_{3}^{l}\}}{1/\phi_{3}(t_{0})+a_{33}^{m}\int_{0}^{t}\exp\{[-r_{3}^{m}+a_{32}^{l}m_{2}/(1+\beta_{2}^{m}M_{2}+\gamma_{2}^{m}M_{3})-0.5{\left(\sigma_{3}^{m}\right)}^{2}]s+\sigma_{3}^{l}B_{3}^{l}\}ds}\\ \;\;\;\;\;\;=\frac{-r_{3}^{m}+a_{32}^{l}m_{2}/(1+\beta_{2}^{m}M_{2}+\gamma_{2}^{m}M_{3})-0.5{\left(\sigma_{3}^{l}\right)}^{2}}{a_{33}^{m}}=m_{3}. \end{array}$$

According to Equations (5)–(11), Theorem 2 is obtained. ☐

## 4. Global Asymptotical Stability of Model (2)

**Definition 2.**  *Model (2) is said to be globally asymptotically stable, if for two arbitrary positive solutions* *$X(t)=(x_{1}(t),x_{2}(t),x_{3}(t))$ and $Y(t)=(y_{1}(t),y_{2}(t),y_{3}(t))$ of model (2), one has*

$$\underset{t\to \infty }{\lim}\left|x_{i}(t)-y_{i}(t)\right|=0,\;(i=1,2,3),\;\;a.s..$$

**Lemma 3.**  *Let $\left(x_{1}(t),x_{2}(t),x_{3}(t)\right)$ be a solution to model (2) with initial conditions (3), then for all p>1, there exists* L(p),G(p)*, and F(p) such that*

$$E\left[x_{1}^{p}(t)\right]\leq L(p),\;E\left[x_{2}^{p}(t)\right]\leq G(p),\;E\left[x_{3}^{p}(t)\right]\leq F(p).$$

**Proof.**  Refer the method of [25] to prove Lemma 3. Define $W(x_{1}(t))=x_{1}^{p}(t)$ for $x_{1}(t)\in R^{+},$ where p>1. By Itô’s formula, we have

$$\begin{array}{l} dW(x_{1}(t))=px_{1}^{p-1}(t)dx_{1}(t)+0.5p(p-1)x_{1}^{p-2}(t){\left(dx_{1}(t)\right)}^{2}\\ \;\;\;\;\;\;\;=px_{1}^{p}(t)[r_{1}(t)-a_{11}(t)x_{1}(t)-\frac{a_{12}(t)x_{2}(t)}{1+\beta_{1}(t)x_{1}(t)+\gamma_{1}(t)x_{2}(t)}\\ \;\;\;\;\;\;\;\;+0.5(p-1)\sigma_{1}^{2}(t)]dt+px_{1}^{p}(t)\sigma_{1}(t)dB_{1}(t). \end{array}$$

Making use of Itô’s formula again for $e^{t}W(x_{1}(t)),$ it follows that

$$\begin{array}{l} d[e^{t}W(x_{1}(t))]=e^{t}W(x_{1}(t))dt+e^{t}dW(x_{1}(t))\\ \;\;\;\;\;\;\;=e^{t}x_{1}^{p}(t)dt+pe^{t}x_{1}^{p}[r_{1}(t)-a_{11}(t)x_{1}(t)-\frac{a_{12}(t)x_{2}(t)}{1+\beta_{1}(t)x_{1}(t)+\gamma_{1}(t)x_{2}(t)}\\ \;\;\;\;\;\;\;\;\;\;+0.5(p-1)\sigma_{1}^{2}(t)]dt\;\;+\;pe^{t}x_{1}^{p}(t)\sigma_{1}(t)dB_{1}(t). \end{array}$$

Integrating both sides of the above inequality from 0 to *t*, we get

$$\begin{array}{l} e^{t}W(x_{1}(t))=x_{1}^{p}(0)+p\int_{0}^{t}e^{s}x_{1}^{p}(s)[1/p+r_{1}(s)-a_{11}(s)x_{1}(s)-\frac{a_{12}(s)x_{2}(s)}{1+\beta_{1}(s)x_{1}(s)+\gamma_{1}(s)x_{2}(s)}\\ \;\;\;\;\;\;\;+0.5(p-1)\sigma_{1}^{2}(s)]ds+p\int_{0}^{t}e^{s}x_{1}^{p}(s)\sigma_{1}(s)dB_{1}(s)\\ \;\;\;\;\;\leq x_{1}^{p}(0)+p\int_{0}^{t}e^{s}x_{1}^{p}(s)[1/p+r_{1}^{m}+0.5p{\left(\sigma_{1}^{m}\right)}^{2}-a_{11}^{l}x_{1}(s)]ds+p\int_{0}^{t}e^{s}x_{1}^{p}(s)\sigma_{1}(s)dB_{1}(s). \end{array}$$

Taking expectations on both sides of the above inequality, we can obtain that

(12) $$\begin{array}{l} E[e^{t}W(x_{1}(t))]\leq x_{1}^{p}(0)+pE\int_{0}^{t}e^{s}\{x_{1}^{p}(s)[1/p+r_{1}^{m}+0.5p{\left(\sigma_{1}^{m}\right)}^{2}-a_{11}^{l}x_{1}(s)]\}ds\\ \;\;\;\;\;\;\;\leq x_{1}^{p}(0)+\int_{0}^{t}e^{s}L_{1}(p)ds=x_{1}^{p}(0)+L_{1}(p)(e^{t}-1), \end{array}$$

where

(13) $$L_{1}(p)=\frac{\left[1+r_{1}^{m}p+0.5p^{2}{\left(\sigma_{1}^{m}\right)}^{2}\right]p+1}{{\left(p+1\right)}^{p+1}{\left(a_{11}^{l}\right)}^{p}}.$$

Define $W(x_{2}(t))=x_{2}^{p}(t),W(x_{3}(t))=x_{3}^{p}(t)$ for $x_{2}(t),x_{3}(t)\in R^{+}$; similarly, we have

(14) $$\begin{array}{l} E[e^{t}W(x_{2}(t))]\leq x_{2}^{p}(0)+pE\int_{0}^{t}e^{s}\{x_{2}^{p}(s)[1/p-r_{2}^{l}+\frac{a_{21}^{m}}{\beta_{1}^{l}}+0.5p{\left(\sigma_{2}^{m}\right)}^{2}-a_{22}^{l}x_{2}(s)]\}ds\\ \;\;\;\;\;\;\leq x_{2}^{p}(0)+\int_{0}^{t}e^{s}G_{1}(p)ds=x_{2}^{p}(0)+G_{1}(p)(e^{t}-1), \end{array}$$

(15) $$\begin{array}{l} E[e^{t}W(x_{3}(t))]\leq x_{3}^{p}(0)+pE\int_{0}^{t}e^{s}\{x_{3}^{p}(s)[1/p-r_{3}^{l}+\frac{a_{32}^{m}}{\beta_{2}^{l}}+0.5p{\left(\sigma_{3}^{m}\right)}^{2}-a_{33}^{l}x_{3}(s)]\}ds\\ \;\;\;\;\;\;\leq x_{3}^{p}(0)+\int_{0}^{t}e^{s}F_{1}(p)ds=x_{3}^{p}(0)+F_{1}(p)(e^{t}-1), \end{array}$$

where

(16) $$G_{1}(p)=\frac{[1-r_{2}^{l}p+(a_{21}^{m}/\beta_{1}^{l})p+0.5p^{2}{\left(\sigma_{2}^{m}\right)}^{2}{]}^{p+1}}{{\left(p+1\right)}^{p+1}{\left(a_{22}^{l}\right)}^{p}},\;F_{1}(p)=\frac{{\left[1-r_{3}^{l}p+(a_{32}^{m}/\beta_{2}^{l})p+0.5p^{2}{\left(\sigma_{3}^{m}\right)}^{2}\right]}^{p+1}}{{\left(p+1\right)}^{p+1}{\left(a_{33}^{l}\right)}^{p}}.$$

Therefore, there exists a T>0 such that $E(x_{1}^{p}(t))\leq 1.5L_{1}(p),\;E(x_{2}^{p}(t))\leq 1.5G_{1}(p)$ and $E(x_{3}^{p}(t))\leq 1.5F_{1}(p)$ for all t≥T. At the same time, an application of the continuity of $E(x_{1}^{p}(t)),E(x_{2}^{p}(t)),E(x_{3}^{p}(t))$ results in that there exist ${\tilde{L}}_{1}(p)>0,{\tilde{G}}_{1}(p)>0,{\tilde{F}}_{1}(p)>0$ such that $E(x_{1}^{p}(t))\leq {\tilde{L}}_{1}(p),E(x_{2}^{p}(t))\leq {\tilde{G}}_{1}(p),E(x_{3}^{p}(t))\leq {\tilde{F}}_{1}(p),$ for t≤T. Let $L(p)=\max\{1.5L_{1}(p),{\tilde{L}}_{1}(p)\},$$G(p)=\max\{1.5G_{1}(p),{\tilde{G}}_{1}(p)\}$ and $F(p)=\max\{1.5F_{1}(p),{\tilde{F}}_{1}(p)\},$ then for all t≥0, we have

(17) $$E\left[x_{1}^{p}(t)\right]\leq L(p),\;E\left[x_{2}^{p}(t)\right]\leq G(p),\;E\left[x_{3}^{p}(t)\right]\leq F(p).$$

According to Equation (17), Lemma 3 holds true. ☐

**Lemma 4**  ([29] p. 54)**.** *Suppose that an n- dimensional stochastic process X={X(t);  0≤t≤T} on a probability space (Ω,F,P) satisfies the condition*

$$E{\left|X(t)-X(s)\right|}^{\alpha_{1}}\leq c{\left|t-s\right|}^{1+\alpha_{2}},\;0\leq s,\;t\leq T,$$

*for some positive constants* $\alpha_{1},\alpha_{2}$*, and* c. *Then there exists a continuous modification* $\tilde{X}=\{\tilde{X}(t);\;0\leq t\leq T\}$ *of* X*, which is locally* $H\ddot{o}lder-$ *continuous with exponent* ϑ *for every* $\vartheta \in (0,\alpha_{1}/\alpha_{2}),$ *i.e., there is a positive random variable* h(ω) *such that*

$$P\left\{\omega :\underset{0<\left|t-s\right|<h(\omega ),0\leq s,t<\infty }{\sup}\frac{\left|\tilde{X}(t,\omega )-\tilde{X}(s,\omega )\right|}{{\left|t-s\right|}^{\vartheta }}\leq \frac{2}{1-2^{-\vartheta }}\right\}=1.$$

*In other words, almost every sample path of* $\tilde{X}$ *is locally but uniformly* $H\ddot{o}lder-$ *continuous with exponent* ϑ.

**Lemma 5.**  *Let $\left(x_{1}(t),x_{2}(t),x_{3}(t)\right)$ be a solution of model (2) with initial value (3), then almost every sample path of $\left(x_{1}(t),x_{2}(t),x_{3}(t)\right)$ is uniformly continuous on t≥0*.

**Proof.**  Refer to the method of [25] to prove Lemma 5. The second equation of model (2) is equivalent to the following stochastic integral equation

(18) $$\begin{array}{l} x_{2}(t)=x_{2}(0)+\int_{0}^{t}x_{2}(s)\left[-r_{2}(s)+\frac{a_{21}(s)x_{1}(s-\tau_{1})}{1+\beta_{1}(s)x_{1}(s-\tau_{1})+\gamma_{1}(s)x_{2}(s-\tau_{1})}-a_{22}(s)x_{2}(s)\right.\\ \;\;\;\;\left.-\frac{a_{23}(s)x_{3}(s)}{1+\beta_{2}(s)x_{2}(s)+\gamma_{2}(s)x_{3}(s)}\right]ds+\int_{0}^{t}\sigma_{2}(s)x_{2}(s)dB_{2}(s). \end{array}$$

Let $\;\frac{1}{p}=\frac{1}{q}=\frac{1}{2}$, by means of the following inequality

$$\begin{array}{l} |E(xy)|\leq {\left[E|x{|}^{p}\right]}^{\frac{1}{p}}{\left[E|y{|}^{q}\right]}^{\frac{1}{q}}\leq 0.5{\left[E|x{|}^{p}\right]}^{\frac{2}{p}}+0.5{\left[E|y{|}^{q}\right]}^{\frac{2}{q}},\;x,y\in R,\\ |x+y+z{|}^{p}\leq 3^{p-1}(|x{|}^{p}+|y{|}^{p}+|z{|}^{p}),\;x,y,z\in R, \end{array}$$

we have

(19) $$\begin{array}{l} \;\;E{\left|x_{2}(t)\left[-r_{2}(t)+\frac{a_{21}(t)x_{1}(t-\tau_{1})}{1+\beta_{1}(t)x_{1}(t-\tau_{1})+\gamma_{1}(t)x_{2}(t-\tau_{1})}-a_{22}(t)x_{2}(t)-\frac{a_{23}(t)x_{3}(t)}{1+\beta_{2}(t)x_{2}(t)+\gamma_{2}(t)x_{3}(t)}\right]\;\right|}^{p}\\ \;\;\;\;\;\leq E\left[{\left|x_{2}(t)\right|}^{p}{\left|-r_{2}(t)+\frac{a_{21}(t)}{\beta_{1}(t)}-a_{22}(t)x_{2}(t)\right|}^{p}\right]\\ \;\;\;\;\;\leq {\left\{E{\left[{\left|x_{2}(t)\right|}^{p}\right]}^{2}\right\}}^{\frac{1}{2}}{\left\{E{\left[{\left|-r_{2}(t)+\frac{a_{21}(t)}{\beta_{1}(t)}-a_{22}(t)x_{2}(t)\right|}^{p}\right]}^{2}\right\}}^{\frac{1}{2}}\\ \;\;\;\;\;\leq 0.5E{\left|x_{2}(t)\right|}^{2p}+0.5E{\left|-r_{2}(t)+\frac{a_{21}(t)}{\beta_{1}(t)}-a_{22}(t)x_{2}(t)\right|}^{2p}\\ \;\;\;\;\;\leq 0.5\{E|x_{2}(t){|}^{2p}+3^{2p-1}[{\left(r_{2}^{m}\right)}^{2p}+{\left(\frac{a_{21}^{m}}{\beta_{1}^{l}}\right)}^{2p}+{\left(a_{22}^{m}\right)}^{2p}E|x_{2}(t){|}^{2p}]\}\\ \;\;\;\;\;\leq 0.5\{G(2p)+3^{2p-1}[{\left(r_{2}^{m}\right)}^{2p}+{\left(\frac{a_{21}^{m}}{\beta_{1}^{l}}\right)}^{2p}+{\left(a_{22}^{m}\right)}^{2p}G(2p)]\}=\;:K_{1}(p). \end{array}$$

Moreover, by the moment inequality for stochastic integral [17] (p. 39), we see that for $0\leq t_{1}\leq t_{2}$ and p>2,

(20) $$\begin{array}{l} \;E{\left|\int_{t_{1}}^{t_{2}}\sigma_{2}(s)x_{2}(s)dB_{2}(s)\right|}^{p}\leq {\left(\sigma_{2}^{m}\right)}^{p}{\left[\frac{p(p-1)}{2}\right]}^{\frac{p}{2}}{\left(t_{2}-t_{1}\right)}^{\frac{p-2}{2}}\int_{t_{1}}^{t_{2}}E|x_{2}(s){|}^{p}ds\\ \;\;\;\;\;\;\;\;\;\;\;\;\leq {\left(\sigma_{2}^{m}\right)}^{p}{\left[\frac{p(p-1)}{2}\right]}^{\frac{p}{2}}{\left(t_{2}-t_{1}\right)}^{\frac{p}{2}}G(p). \end{array}$$

Thus, for $0\leq t_{1}\leq t_{2}<+\infty ,t_{2}-t_{1}\leq 1,\frac{1}{p}+\frac{1}{q}=1,$ by the elementary inequality $|x+y{|}^{p}\leq 2^{p-1}(|x{|}^{p}$ $+|y{|}^{p}),\;x,y\in R$, $H\ddot{o}lder$ inequality [17] (p. 61), and (18)–(20), it is easy to see that

(21) $$\begin{array}{l} E\left({\left|x_{2}(t_{2})-x_{2}(t_{1})\right|}^{p}\right)=E\left|\int_{t_{1}}^{t_{2}}x_{2}(s)[-r_{2}(s)+\frac{a_{21}(s)x_{1}(s-\tau_{1})}{1+\beta_{1}(s)x_{1}(s-\tau_{1})+\gamma_{1}(s)x_{2}(s-\tau_{1})}-a_{22}(s)x_{2}(s)\right.\\ \;\;\;\;\;\;\;\;\;-\frac{a_{23}(s)x_{3}(s)}{1+\beta_{2}(s)x_{2}(s)+\gamma_{2}(s)x_{3}(s)}]ds+{\left.\int_{t_{1}}^{t_{2}}\sigma_{2}(s)x_{2}(s)dB_{2}(s)\right|}^{p}\\ \;\;\;\;\;\;\;\;\leq 2^{p-1}E\left|\int_{t_{1}}^{t_{2}}x_{2}(s)\left[-r_{2}(s)+\frac{a_{21}(s)x_{1}(s-\tau_{1})}{1+\beta_{1}(s)x_{1}(s-\tau_{1})+\gamma_{1}(s)x_{2}(s-\tau_{1})}-a_{22}(s)x_{2}(s)\right.\right.\\ {\;\left.\;\;\;\;\;\;\;\;-\frac{a_{23}(s)x_{3}(s)}{1+\beta_{2}(s)x_{2}(s)+\gamma_{2}(s)x_{3}(s)}\right]ds|}^{p}+2^{p-1}E{\left|\int_{t_{1}}^{t_{2}}\sigma_{2}(s)x_{2}(s)dB_{2}(s)\right|}^{p}\\ \;\;\;\;\;\;\;\;\leq 2^{p-1}{\left(t_{2}-t_{1}\right)}^{\frac{p}{q}}\int_{t_{1}}^{t_{2}}E\left|x_{2}(s)\left[-r_{2}(s)+\frac{a_{21}(s)x_{1}(s-\tau_{1})}{1+\beta_{1}(s)x_{1}(s-\tau_{1})+\gamma_{1}(s)x_{2}(s-\tau_{1})}-a_{22}(s)x_{2}(s)\right.\right.\\ {\;\;\;\;\;\;\;\;\;\left.-\frac{a_{23}(s)x_{3}(s)}{1+\beta_{2}(s)x_{2}(s)+\gamma_{2}(s)x_{3}(s)}\right]}^{p}ds+2^{p-1}{\left[\frac{p(p-1)}{2}\right]}^{\frac{p}{2}}{\left(t_{2}-t_{1}\right)}^{\frac{p}{2}}{\left(\sigma_{2}^{m}\right)}^{p}G(p)\\ \;\;\;\;\;\;\;\;\leq 2^{p-1}{\left(t_{2}-t_{1}\right)}^{\frac{p+q}{q}}K_{1}(p)+2^{p-1}{\left[\frac{p(p-1)}{2}\right]}^{\frac{p}{2}}{\left(t_{2}-t_{1}\right)}^{\frac{p}{2}}{\left(\sigma_{2}^{m}\right)}^{p}G(p)\\ \;\;\;\;\;\;\;\;\leq 2^{p-1}{\left(t_{2}-t_{1}\right)}^{\frac{p}{2}}\left[{\left(t_{2}-t_{1}\right)}^{\frac{p}{2}}+{\left[\frac{p(p-1)}{2}\right]}^{\frac{p}{2}}\right]K_{2}(p)\\ \;\;\;\;\;\;\;\;\leq 2^{p-1}{\left(t_{2}-t_{1}\right)}^{\frac{p}{2}}\left[1+{\left[\frac{p(p-1)}{2}\right]}^{\frac{p}{2}}\right]K_{2}(p), \end{array}$$

where $K_{2}(p)=\max\{K_{1}(p),{\left(\sigma_{2}^{m}\right)}^{p}G(p)\}$. Then it follows from Lemma 4 that almost every sample path of $x_{2}(t)$ is locally but uniformly $H\ddot{o}lder-$ continuous with exponent ϑ for every $\vartheta \in \left(0,\frac{p-2}{2p}\right)$, and therefore, almost every sample path of $x_{2}(t)$ is uniformly continuous. Similarly, we can obtain that every sample path of $x_{1}(t)$ and $x_{3}(t)$ are uniformly continuous. ☐

**Lemma 6**  ([30] Lemma 8.2)**.** *Let f(t) be a non-negative continuous function on the interval* [0,+∞)*; if it is integrable on the interval [0,+∞) and is uniformly continuous, then $\underset{t\to \infty }{\lim}\;f(t)=0$.*

**Theorem 3.**  *Assume that model (2) satisfies $\left(H_{1})-(H_{6}\right)$ and the following conditions*

$$(H_{7})\;A>0,\;B>0,\;C>0,$$

*where*

$$A=\lambda_{1}a_{11}^{l}-\frac{\lambda_{1}a_{12}^{m}\beta_{1}^{m}M_{2}}{{\left(1+\beta_{1}^{l}m_{1}+\gamma_{1}^{l}m_{2}\right)}^{2}}-\frac{\lambda_{2}(a_{21}^{m}+a_{21}^{m}\gamma_{1}^{m}M_{2})}{{\left(1+\beta_{1}^{l}m_{1}+\gamma_{1}^{l}m_{2}\right)}^{2}},$$

$$B=\lambda_{2}a_{22}^{l}-\frac{\lambda_{1}(a_{12}^{m}+a_{12}^{m}\beta_{1}^{m}M_{1})}{{\left(1+\beta_{1}^{l}m_{1}+\gamma_{1}^{l}m_{2}\right)}^{2}}-\frac{\lambda_{2}a_{23}^{m}\beta_{2}^{m}M_{3}}{{\left(1+\beta_{2}^{l}m_{2}+\gamma_{2}^{l}m_{3}\right)}^{2}}-\frac{\lambda_{2}a_{21}^{m}\gamma_{1}^{m}M_{1}}{{\left(1+\beta_{1}^{l}m_{1}+\gamma_{1}^{l}m_{2}\right)}^{2}}-\frac{\lambda_{3}(a_{32}^{m}+a_{32}^{m}\gamma_{2}^{m}M_{3})}{{\left(1+\beta_{2}^{l}m_{2}+\gamma_{2}^{l}m_{3}\right)}^{2}},$$

$$C=\lambda_{3}a_{33}^{l}-\frac{\lambda_{2}(a_{23}^{m}+a_{23}^{m}\beta_{2}^{m}M_{2})}{{\left(1+\beta_{2}^{l}m_{2}+\gamma_{2}^{l}m_{3}\right)}^{2}}-\frac{\lambda_{3}a_{32}^{m}\gamma_{2}^{m}M_{2}}{{\left(1+\beta_{2}^{l}m_{2}+\gamma_{2}^{l}m_{3}\right)}^{2}},\lambda_{i}\;(i=1,2,3)\;\mathrm{are}\;\mathrm{positive}\;\mathrm{constraints}$$

*Then model (2) is globally asymptotically stable.*

**Proof.**  For any two positive solutions $\left(x_{1}(t),x_{2}(t),x_{3}(t)\right)$ and $\left(y_{1}(t),y_{2}(t),y_{3}(t)\right)$ of model (2), by Theorem 3.1, there exists positive number $m_{i},M_{i}(i=1,2,3)$ and T, such that

$$m_{i}\leq x_{i}(t)\leq M_{i},\;m_{i}\leq y_{i}(t)\leq M_{i},(i=1,2,3),\;t\geq T.$$

Constructing a Lyapunov function

(22) $$V(t)=V_{1}(t)+V_{2}(t)+V_{3}(t)+V_{4}(t)+V_{5}(t),$$

where

$$\begin{array}{l} V_{1}(t)=\lambda_{1}\left|\ln x_{1}(t)-\ln y_{1}(t)\right|+\lambda_{2}\left|\ln x_{2}(t)-\ln y_{2}(t)\right|+\lambda_{3}\left|\ln x_{3}(t)-\ln y_{3}(t)\right|,\\ V_{2}(t)=\frac{\lambda_{2}(a_{21}^{m}+a_{21}^{m}\gamma_{1}^{m}M_{2})}{{\left(1+\beta_{1}^{l}m_{1}+\gamma_{1}^{l}m_{2}\right)}^{2}}\int_{t-\tau_{1}}^{t}\left|x_{1}(s)-y_{1}(s)\right|ds,\\ V_{3}(t)=\frac{\lambda_{2}a_{21}^{m}\gamma_{1}^{m}M_{1}}{{\left(1+\beta_{1}^{l}m_{1}+\gamma_{1}^{l}m_{2}\right)}^{2}}\int_{t-\tau_{1}}^{t}\left|x_{2}(s)-y_{2}(s)\right|ds,\\ V_{4}(t)=\frac{\lambda_{3}(a_{32}^{m}+a_{32}^{m}\gamma_{2}^{m}M_{3})}{{\left(1+\beta_{2}^{l}m_{2}+\gamma_{2}^{l}m_{3}\right)}^{2}}\int_{t-\tau_{2}}^{t}\left|x_{2}(s)-x_{2}(s)\right|ds,\\ V_{5}(t)=\frac{\lambda_{3}a_{32}^{m}\gamma_{2}^{m}M_{2}}{{\left(1+\beta_{2}^{l}m_{2}+\gamma_{2}^{l}m_{3}\right)}^{2}}\int_{t-\tau_{2}}^{t}\left|x_{3}(s)-y_{3}(s)\right|ds. \end{array}$$

Calculating the upper right derivative of $d^{+}V_{1}(t)$ along model (2), we have

$$\begin{array}{l} d^{+}V_{1}(t)=\lambda_{1}\mathrm{sgn}[x_{1}(t)-y_{1}(t)]\left\{\left[\frac{dx_{1}}{x_{1}}-\frac{{\left(dx_{1}\right)}^{2}}{2x_{1}^{2}}\right]-\left[\frac{dy_{1}}{y_{1}}-\frac{{\left(dy_{1}\right)}^{2}}{2y_{1}^{2}}\right]\right\}\\ \;\;\;\;\;+\lambda_{2}\mathrm{sgn}[x_{2}(t)-y_{2}(t)]\left\{\left[\frac{dx_{2}}{x_{2}}-\frac{{\left(dx_{2}\right)}^{2}}{2x_{2}^{2}}\right]-\left[\frac{dy_{2}}{y_{2}}-\frac{{\left(dy_{2}\right)}^{2}}{2y_{2}^{2}}\right]\right\}\\ \;\;\;\;\;\;+\lambda_{3}\mathrm{sgn}[x_{3}(t)-y_{3}(t)]\left\{\left[\frac{dx_{3}}{x_{3}}-\frac{{\left(dx_{3}\right)}^{2}}{2x_{3}^{2}}\right]-\left[\frac{dy_{3}}{y_{3}}-\frac{{\left(dy_{3}\right)}^{2}}{2y_{3}^{2}}\right]\right\}\\ =\lambda_{1}\mathrm{sgn}[x_{1}(t)-y_{1}(t)]\left\{-a_{11}(t)(x_{1}(t)-y_{1}(t))-\left(\frac{a_{12}(t)x_{2}(t)}{1+\beta_{1}(t)x_{1}(t)+\gamma_{1}(t)x_{2}(t)}-\frac{a_{12}(t)y_{2}(t)}{1+\beta_{1}(t)y_{1}(t)+\gamma_{1}(t)y_{2}(t)}\right)\right\}dt\\ \;+\lambda_{2}\mathrm{sgn}[x_{2}(t)-y_{2}(t)]\left\{-a_{22}(t)(x_{2}(t)-y_{2}(t))\right.-\left(\frac{a_{23}(t)x_{3}(t)}{1+\beta_{2}(t)x_{2}(t)+\gamma_{2}(t)x_{3}(t)}-\frac{a_{23}(t)y_{3}(t)}{1+\beta_{2}(t)y_{2}(t)+\gamma_{2}(t)y_{3}(t)}\right)\\ \;\left.+\left(\frac{a_{21}(t)x_{1}(t-\tau_{1})}{1+\beta_{1}(t)x_{1}(t-\tau_{1})+\gamma_{1}(t)x_{2}(t-\tau_{1})}-\frac{a_{21}(t)y_{1}(t-\tau_{1})}{1+\beta_{1}(t)y_{1}(t-\tau_{1})+\gamma_{1}(t)y_{2}(t-\tau_{1})}\right)\right\}dt\\ \;+\lambda_{3}\mathrm{sgn}[x_{3}(t)-y_{3}(t)]\left\{-a_{33}(t)(x_{3}(t)-y_{3}(t))\right.\\ +\left.\left(\frac{a_{32}(t)x_{2}(t-\tau_{2})}{1+\beta_{2}(t)x_{2}(t-\tau_{2})+\gamma_{2}(t)x_{3}(t-\tau_{2})}-\frac{a_{32}(t)y_{2}(t-\tau_{2})}{1+\beta_{2}(t)y_{2}(t-\tau_{2})+\gamma_{2}(t)y_{3}(t-\tau_{2})}\right)\right\}dt \end{array}$$

$$\begin{array}{l} =\lambda_{1}\mathrm{sgn}[x_{1}(t)-y_{1}(t)]\left\{-\left(\frac{\left(a_{12}(t)+a_{12}(t)\beta_{1}(t)y_{1}(t))(x_{2}(t)-y_{2}(t))-a_{12}(t)\beta_{1}(t)y_{2}(t)(x_{1}(t)-y_{1}(t)\right)}{\left(1+\beta_{1}(t)x_{1}(t)+\gamma_{1}(t)x_{2}(t))(1+\beta_{1}(t)y_{1}(t)+\gamma_{1}(t)y_{2}(t)\right)}\right)\right.\\ \;\left.-a_{11}(t)(x_{1}(t)-y_{1}(t))\right\}dt+\lambda_{2}\mathrm{sgn}[x_{2}(t)-y_{2}(t)]\left\{-a_{22}(t)(x_{2}(t)-y_{2}(t))\right.\\ \;+\left(\frac{\left(a_{21}(t)+a_{21}(t)\gamma_{1}(t)y_{2}(t-\tau_{1}))(x_{1}(t-\tau_{1})-y_{1}(t-\tau_{1}))-a_{21}(t)\gamma_{1}(t)y_{1}(t-\tau_{1})(x_{2}(t-\tau_{1})-y_{2}(t-\tau_{1})\right)}{\left(1+\beta_{1}(t)x_{1}(t-\tau_{1})+\gamma_{1}(t)x_{2}(t-\tau_{1}))(1+\beta_{1}(t)x_{1}(t-\tau_{1})+\gamma_{1}(t)x_{2}(t-\tau_{1})\right)}\right)\\ \left.\;-\left(\frac{\left(a_{23}(t)+a_{23}(t)\beta_{2}(t)y_{2}(t))(x_{3}(t)-y_{3}(t))-a_{23}(t)\beta_{2}(t)y_{3}(t)(x_{2}(t)-y_{2}(t)\right)}{\left(1+\beta_{2}(t)x_{2}(t)+\gamma_{2}(t)x_{3}(t))(1+\beta_{2}(t)y_{2}(t)+\gamma_{2}(t)y_{3}(t)\right)}\right)\right\}dt\\ \;+\lambda_{3}\mathrm{sgn}[x_{3}(t)-y_{3}(t)]\left\{-a_{33}(t)(x_{3}(t)-y_{3}(t))\right.\\ \;\left.+\left(\frac{\left(a_{32}(t)+a_{32}(t)\gamma_{2}(t)y_{3}(t-\tau_{2}))(x_{2}(t-\tau_{2})-y_{2}(t-\tau_{2}))-a_{32}(t)\gamma_{2}(t)y_{2}(t-\tau_{2})(x_{3}(t-\tau_{2})-y_{3}(t-\tau_{2})\right)}{\left(1+\beta_{2}(t)x_{2}(t-\tau_{2})+\gamma_{2}(t)x_{3}(t-\tau_{2}))(1+\beta_{2}(t)y_{2}(t-\tau_{2})+\gamma_{2}(t)y_{3}(t-\tau_{2})\right)}\right)\right\}dt \end{array}$$

(23) $$\begin{array}{l} \;\leq \left\{\frac{\lambda_{1}(a_{12}(t)+a_{12}(t)\beta_{1}(t)y_{1}(t))}{\left(1+\beta_{1}(t)x_{1}(t)+\gamma_{1}(t)x_{2}(t))(1+\beta_{1}(t)y_{1}(t)+\gamma_{1}(t)y_{2}(t)\right)}\left|x_{2}(t)-y_{2}(t)\right|\right.\\ +\frac{\lambda_{1}a_{12}(t)\beta_{1}(t)y_{2}(t)}{\left(1+\beta_{1}(t)x_{1}(t)+\gamma_{1}(t)x_{2}(t))(1+\beta_{1}(t)y_{1}(t)+\gamma_{1}(t)y_{2}(t)\right)}\left|x_{1}(t)-y_{1}(t)\right|\\ -\lambda_{1}a_{11}(t)\left|x_{1}(t)-y_{1}(t)\right|-\lambda_{2}a_{22}(t)\left|x_{2}(t)-y_{2}(t)\right|\\ +\frac{\lambda_{2}(a_{21}(t)+a_{21}(t)\gamma_{1}(t)y_{2}(t-\tau_{1}))}{\left(1+\beta_{1}(t)x_{1}(t-\tau_{1})+\gamma_{1}(t)x_{2}(t-\tau_{1}))(1+\beta_{1}(t)x_{1}(t-\tau_{1})+\gamma_{1}(t)x_{2}(t-\tau_{1})\right)}\left|x_{1}(t-\tau_{1})-y_{1}(t-\tau_{1})\right|\\ +\frac{\lambda_{2}a_{21}(t)\gamma_{1}(t)y_{1}(t-\tau_{1})}{\left(1+\beta_{1}(t)x_{1}(t-\tau_{1})+\gamma_{1}(t)x_{2}(t-\tau_{1}))(1+\beta_{1}(t)x_{1}(t-\tau_{1})+\gamma_{1}(t)x_{2}(t-\tau_{1})\right)}\left|x_{2}(t-\tau_{1})-y_{2}(t-\tau_{1})\right|\\ +\frac{\lambda_{2}(a_{23}(t)+a_{23}(t)\beta_{2}(t)y_{2}(t))}{\left(1+\beta_{2}(t)x_{2}(t)+\gamma_{2}(t)x_{3}(t))(1+\beta_{2}(t)y_{2}(t)+\gamma_{2}(t)y_{3}(t)\right)}\left|x_{3}(t)-y_{3}(t)\right|\\ +\frac{\lambda_{2}a_{23}(t)\beta_{2}(t)y_{3}(t)}{\left(1+\beta_{2}(t)x_{2}(t)+\gamma_{2}(t)x_{3}(t))(1+\beta_{2}(t)y_{2}(t)+\gamma_{2}(t)y_{3}(t)\right)}\left|x_{2}(t)-y_{2}(t)\right|-\lambda_{3}a_{33}(t)\left|x_{3}(t)-y_{3}(t)\right|\\ +\frac{\lambda_{3}(a_{32}(t)+a_{32}(t)\gamma_{2}(t)y_{3}(t-\tau_{2}))}{\left(1+\beta_{2}(t)x_{2}(t-\tau_{2})+\gamma_{2}(t)x_{3}(t-\tau_{2}))(1+\beta_{2}(t)y_{2}(t-\tau_{2})+\gamma_{2}(t)y_{3}(t-\tau_{2})\right)}\left|x_{2}(t-\tau_{2})-y_{2}(t-\tau_{2})\right|\\ \left.\frac{\lambda_{3}a_{32}(t)\gamma_{2}(t)y_{2}(t-\tau_{2})}{\left(1+\beta_{2}(t)x_{2}(t-\tau_{2})+\gamma_{2}(t)x_{3}(t-\tau_{2}))(1+\beta_{2}(t)y_{2}(t-\tau_{2})+\gamma_{2}(t)y_{3}(t-\tau_{2})\right)}\left|x_{3}(t-\tau_{2})-y_{3}(t-\tau_{2})\right|\right\}dt. \end{array}$$

Calculating the upper right derivative of $d^{+}V_{i}(t)(i=2,3,4,5)$ along system (1.2), we have

(24) $$d^{+}V_{2}(t)=\frac{\lambda_{2}(a_{21}^{m}+a_{21}^{m}\gamma_{1}^{m}M_{2})}{{\left(1+\beta_{1}^{l}m_{1}+\gamma_{1}^{l}m_{2}\right)}^{2}}\left|x_{1}(t)-y_{1}(t)\right|dt-\frac{\lambda_{2}(a_{21}^{m}+a_{21}^{m}\gamma_{1}^{m}M_{2})}{{\left(1+\beta_{1}^{l}m_{1}+\gamma_{1}^{l}m_{2}\right)}^{2}}\left|x_{1}(t-\tau_{1})-y_{1}(t-\tau_{1})\right|dt,$$

(25) $$d^{+}V_{3}(t)=\frac{\lambda_{2}a_{21}^{m}\gamma_{1}^{m}M_{1}}{{\left(1+\beta_{1}^{l}m_{1}+\gamma_{1}^{l}m_{2}\right)}^{2}}\left|x_{2}(t)-y_{2}(t)\right|dt-\frac{\lambda_{2}a_{21}^{m}\gamma_{1}^{m}M_{1}}{{\left(1+\beta_{1}^{l}m_{1}+\gamma_{1}^{l}m_{2}\right)}^{2}}\left|x_{2}(t-\tau_{1})-y_{2}(t-\tau_{1})\right|dt,$$

(26) $$d^{+}V_{4}(t)=\frac{\lambda_{3}(a_{32}^{m}+a_{32}^{m}\gamma_{2}^{m}M_{3})}{{\left(1+\beta_{2}^{l}m_{2}+\gamma_{2}^{l}m_{3}\right)}^{2}}\left|x_{2}(t)-y_{2}(t)\right|dt-\frac{\lambda_{3}(a_{32}^{m}+a_{32}^{m}\gamma_{2}^{m}M_{3})}{{\left(1+\beta_{2}^{l}m_{2}+\gamma_{2}^{l}m_{3}\right)}^{2}}\left|x_{2}(t-\tau_{2})-y_{2}(t-\tau_{2})\right|dt,$$

(27) $$d^{+}V_{5}(t)=\frac{\lambda_{3}a_{32}^{m}\gamma_{2}^{m}M_{2}}{{\left(1+\beta_{2}^{l}m_{2}+\gamma_{2}^{l}m_{3}\right)}^{2}}\left|x_{3}(t)-y_{3}(t)\right|dt-\frac{\lambda_{3}a_{32}^{m}\gamma_{2}^{m}M_{2}}{{\left(1+\beta_{2}^{l}m_{2}+\gamma_{2}^{l}m_{3}\right)}^{2}}\left|x_{3}(t-\tau_{2})-y_{3}(t-\tau_{2})\right|dt.$$

Combining Equations (22)–(27), we have

$$\begin{array}{l} d^{+}V(t)\leq \left\{\frac{\lambda_{1}(a_{12}^{m}+a_{12}^{m}\beta_{1}^{m}M_{1})}{{\left(1+\beta_{1}^{l}m_{1}+\gamma_{1}^{l}m_{2}\right)}^{2}}\left|x_{2}(t)-y_{2}(t)\right|+\frac{\lambda_{1}a_{12}^{m}\beta_{1}^{m}M_{2}}{{\left(1+\beta_{1}^{l}m_{1}+\gamma_{1}^{l}m_{2}\right)}^{2}}\left|x_{1}(t)-y_{1}(t)\right|\right.\\ \;-\lambda_{1}a_{11}^{l}\left|x_{1}(t)-y_{1}(t)\right|-\lambda_{2}a_{22}^{l}\left|x_{2}(t)-y_{2}(t)\right|+\frac{\lambda_{2}(a_{21}^{m}+a_{21}^{m}\gamma_{1}^{m}M_{2})}{{\left(1+\beta_{1}^{l}m_{1}+\gamma_{1}^{l}m_{2}\right)}^{2}}\left|x_{1}(t-\tau_{1})-y_{1}(t-\tau_{1})\right|\\ \;+\frac{\lambda_{2}a_{21}^{m}\gamma_{1}^{m}M_{1}}{{\left(1+\beta_{1}^{l}m_{1}+\gamma_{1}^{l}m_{2}\right)}^{2}}\left|x_{2}(t-\tau_{1})-y_{2}(t-\tau_{1})\right|+\frac{\lambda_{2}(a_{23}^{m}+a_{23}^{m}\beta_{2}^{m}M_{2})}{{\left(1+\beta_{2}^{l}m_{2}+\gamma_{2}^{l}m_{3}\right)}^{2}}\left|x_{3}(t)-y_{3}(t)\right|\\ \;+\frac{\lambda_{2}a_{23}^{m}\beta_{2}^{m}M_{3}}{{\left(1+\beta_{2}^{l}m_{2}+\gamma_{2}^{l}m_{3}\right)}^{2}}\left|x_{2}(t)-y_{2}(t)\right|\;-\lambda_{3}a_{33}^{l}\left|x_{3}(t)-y_{3}(t)\right|\\ \;+\frac{\lambda_{3}(a_{32}^{m}+a_{32}^{m}\gamma_{2}^{m}M_{3})}{{\left(1+\beta_{2}^{l}m_{2}+\gamma_{2}^{l}m_{3}\right)}^{2}}\left|x_{2}(t-\tau_{2})-y_{2}(t-\tau_{2})\right|+\frac{\lambda_{3}a_{32}^{m}\gamma_{2}^{m}M_{2}}{{\left(1+\beta_{2}^{l}m_{2}+\gamma_{2}^{l}m_{3}\right)}^{2}}\left|x_{3}(t-\tau_{2})-y_{3}(t-\tau_{2})\right|\\ +\frac{\lambda_{2}(a_{21}^{m}+a_{21}^{m}\gamma_{1}^{m}M_{2})}{{\left(1+\beta_{1}^{l}m_{1}+\gamma_{1}^{l}m_{2}\right)}^{2}}\left|x_{1}(t)-y_{1}(t)\right|-\frac{\lambda_{2}(a_{21}^{m}+a_{21}^{m}\gamma_{1}^{m}M_{2})}{{\left(1+\beta_{1}^{l}m_{1}+\gamma_{1}^{l}m_{2}\right)}^{2}}\left|x_{1}(t-\tau_{1})-y_{1}(t-\tau_{1})\right|\\ +\frac{\lambda_{2}a_{21}^{m}\gamma_{1}^{m}M_{1}}{{\left(1+\beta_{1}^{l}m_{1}+\gamma_{1}^{l}m_{2}\right)}^{2}}\left|x_{2}(t)-y_{2}(t)\right|-\frac{\lambda_{2}a_{21}^{m}\gamma_{1}^{m}M_{1}}{{\left(1+\beta_{1}^{l}m_{1}+\gamma_{1}^{l}m_{2}\right)}^{2}}\left|x_{2}(t-\tau_{1})-y_{2}(t-\tau_{1})\right|\\ +\frac{\lambda_{3}(a_{32}^{m}+a_{32}^{m}\gamma_{2}^{m}M_{3})}{{\left(1+\beta_{2}^{l}m_{2}+\gamma_{2}^{l}m_{3}\right)}^{2}}\left|x_{2}(t)-y_{2}(t)\right|-\frac{\lambda_{3}(a_{32}^{m}+a_{32}^{m}\gamma_{2}^{m}M_{3})}{{\left(1+\beta_{2}^{l}m_{2}+\gamma_{2}^{l}m_{3}\right)}^{2}}\left|x_{2}(t-\tau_{2})-y_{2}(t-\tau_{2})\right|\\ \;\left.+\frac{\lambda_{3}a_{32}^{m}\gamma_{2}^{m}M_{2}}{{\left(1+\beta_{2}^{l}m_{2}+\gamma_{2}^{l}m_{3}\right)}^{2}}\left|x_{3}(t)-y_{3}(t)\right|-\frac{\lambda_{3}a_{32}^{m}\gamma_{2}^{m}M_{2}}{{\left(1+\beta_{2}^{l}m_{2}+\gamma_{2}^{l}m_{3}\right)}^{2}}\left|x_{3}(t-\tau_{2})-y_{3}(t-\tau_{2})\right|\right\}dt\\ \leq \left\{-\left(\lambda_{1}a_{11}^{l}-\frac{\lambda_{1}a_{12}^{m}\beta_{1}^{m}M_{2}}{{\left(1+\beta_{1}^{l}m_{1}+\gamma_{1}^{l}m_{2}\right)}^{2}}-\frac{\lambda_{2}(a_{21}^{m}+a_{21}^{m}\gamma_{1}^{m}M_{2})}{{\left(1+\beta_{1}^{l}m_{1}+\gamma_{1}^{l}m_{2}\right)}^{2}}\right)\left|x_{1}(t)-y_{1}(t)\right|-\left(\lambda_{2}a_{22}^{l}-\frac{\lambda_{1}(a_{12}^{m}+a_{12}^{m}\beta_{1}^{m}M_{1})}{{\left(1+\beta_{1}^{l}m_{1}+\gamma_{1}^{l}m_{2}\right)}^{2}}\right.\right. \end{array}$$

(28) $$\begin{array}{l} \;\left.-\frac{\lambda_{2}a_{23}^{m}\beta_{2}^{m}M_{3}}{{\left(1+\beta_{2}^{l}m_{2}+\gamma_{2}^{l}m_{3}\right)}^{2}}-\frac{\lambda_{2}a_{21}^{m}\gamma_{1}^{m}M_{1}}{{\left(1+\beta_{1}^{l}m_{1}+\gamma_{1}^{l}m_{2}\right)}^{2}}-\frac{\lambda_{3}(a_{32}^{m}+a_{32}^{m}\gamma_{2}^{m}M_{3})}{{\left(1+\beta_{2}^{l}m_{2}+\gamma_{2}^{l}m_{3}\right)}^{2}}\right)\left|x_{2}(t)-y_{2}(t)\right|\\ \;\left.-\left(\lambda_{3}a_{33}^{l}-\frac{\lambda_{2}(a_{23}^{m}+a_{23}^{m}\beta_{2}^{m}M_{2})}{{\left(1+\beta_{2}^{l}m_{2}+\gamma_{2}^{l}m_{3}\right)}^{2}}-\frac{\lambda_{3}a_{32}^{m}\gamma_{2}^{m}M_{2}}{{\left(1+\beta_{2}^{l}m_{2}+\gamma_{2}^{l}m_{3}\right)}^{2}}\right)\left|x_{3}(t)-y_{3}(t)\right|\right\}dt\\ =-A\left|x_{1}(t)-y_{1}(t)\right|dt-B\left|x_{2}(t)-y_{2}(t)\right|dt-C\left|x_{3}(t)-y_{3}(t)\right|dt. \end{array}$$

By the assumption of $\left(H_{7}\right)$ of Theorem 3, there exists α>0 such that

(29) A≥α>0, B≥α>0, C≥α>0.

Integrating Equation (28) from 0 to *t*, we have

(30) $$V(t)+\alpha \int_{0}^{t}\sum_{i=1}^{3}\left|x_{i}(s)-y_{i}(s)\right|ds\leq V(0)<+\infty ,$$

and V(t) is a bounded function in interval [0,t], so there is

(31) $$\alpha \int_{0}^{t}\sum_{i=1}^{3}\left|x_{i}(s)-y_{i}(s)\right|ds<+\infty .$$

By V(t)≥0, we have

(32) $$\sum_{i=1}^{3}\left|x_{i}(t)-y_{i}(t)\right|\in L^{1}[0,+\infty ).$$

Thus, from Lemmas 5 and 6, we get

$$\underset{t\to +\infty }{\lim}\left|x_{i}(t)-y_{i}(t)\right|=0,\;(\;i=1,2,3).$$

That is, model (2) is globally asymptotically stable. ☐

## 5. Numerical Simulation and Biological Interpretation

In this section, MATLAB 2023 simulation software and the Milstein discrete method [31,32,33] are employed to simulate the system. Considering the seasonal influence of weather, both the food supply and mating habits of biological populations exhibit periodic changes over time. Periodic functions are selected as parameters in model (2) to verify the validity of the aforementioned theoretical analysis. The following three-population food chain predation model, which incorporates time delay and the Beddington–DeAngelis functional response, is presented.

(33) $$\left\{\begin{array}{l} \begin{array}{l} dx_{1}(t)=x_{1}(t)\left[\left(5.5+0.5\sin\pi t)-(1.2+1\left|\sin\pi t\right|)x_{1}(t\right)\right.\\ \;\;\;\left.-\frac{\left(0.062+0.0005\sin\pi t)x_{2}(t\right)}{1+(0.5+0.1\sin\pi t)x_{1}(t)+(0.11+0.01\sin\pi t)x_{2}(t)}\right]dt+(0.5+0.1\left|\sin\pi t\right|)x_{1}(t)dB_{1}(t), \end{array}\\ \begin{array}{l} dx_{2}(t)=x_{2}(t)\left[-(0.005+0.001\left|\cos\pi t\right|)+\frac{\left(5.5+0.5\left|\sin\pi t\right|)x_{1}(t-\tau_{1}\right)}{1+(0.5+0.1\sin\pi t)x_{1}(t-\tau_{1})+(0.11+0.01\sin\pi t)x_{2}(t-\tau_{1})}\right.\\ \;\;\;\left.-(2.75+0.25\left|\cos\pi t\right|)x_{2}(t)-\frac{\left(0.03+0.004\sin\pi t)x_{3}(t\right)}{1+(1.5+0.2\left|\sin\pi t\right|)x_{2}(t)+(0.11+0.01\sin\pi t)x_{3}(t)}\right]dt\\ \;\;\;+(0.2+0.1\left|\sin\pi t\right|)x_{2}(t)dB_{2}(t), \end{array}\\ \begin{array}{l} dx_{3}(t)=x_{3}(t)\left[-(0.002+0.001\left|\sin\pi t\right|)+\frac{\left(10+0.5\sin\pi t)x_{2}(t-\tau_{2}\right)}{1+(1.5+0.2\left|\sin\pi t\right|)x_{2}(t-\tau_{2})+(0.11+0.01\sin\pi t)x_{3}(t-\tau_{2})}\right.\\ \;\;\;-(4.25+0.2\left|\cos\pi t\right|)x_{3}(t)]dt+(0.15+0.075\sin\pi t)x_{3}(t)dB_{3}(t). \end{array} \end{array}\right.$$

By simple calculation, we obtain

$$\begin{array}{l} M_{1}=4.90,\;\;M_{2}=5.44,\;\;M_{3}=1.65,\;\;m_{1}=1.91,\;\;m_{2}=0.63,\;\;m_{3}=0.12,\\ r_{1}^{m}-0.5{\left(\sigma_{1}^{l}\right)}^{2}=5.875,\;\;a_{21}^{m}/\beta_{1}^{l}-r_{2}^{l}-0.5{\left(\sigma_{2}^{l}\right)}^{2}=14.976,\;\;a_{32}^{m}/\beta_{2}^{l}-r_{3}^{l}-0.5{\left(\sigma_{3}^{l}\right)}^{2}=6.995,\\ r_{1}^{l}-a_{12}^{m}/\gamma_{1}^{l}-0.5(\sigma_{1}^{m})=4.195,\;\;a_{21}^{l}m_{1}/(1+\beta_{1}^{m}M_{1}+\gamma_{1}^{m}M_{2})-r_{2}^{m}-a_{23}^{m}/\gamma_{2}^{l}-0.5{\left(\sigma_{2}^{m}\right)}^{2}=1.896,\\ a_{32}^{l}m_{2}/(1+\beta_{2}^{m}M_{2}+\gamma_{2}^{m}M_{3})-r_{3}^{m}-0.5{\left(\sigma_{3}^{m}\right)}^{2}=0.545,\;\;A=0.45,\;\;B=0.55,\;\;C=0.40. \end{array}$$

**Figure 1:**
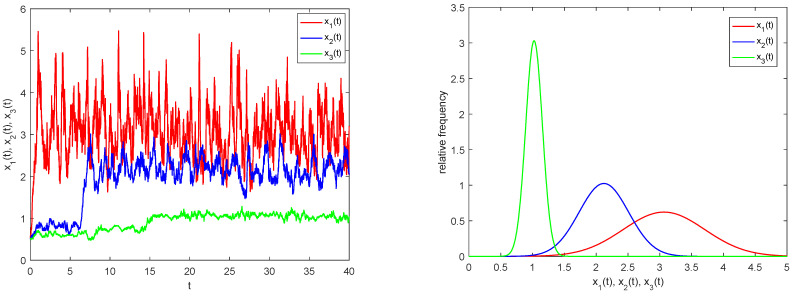
Numerical simulation of models (33)–(34).

It can be observed from the calculation results that the conditions specified in both Theorems 2 and 3 are satisfied, confirming that model (33) demonstrates uniform persistence and global asymptotic stability. By implementing the time delays $\tau_{1}=6,\tau_{2}=8$, step length Δt=0.01, and initial values

(34) $$x_{1}(t_{0})=0.5,\;x_{2}(t_{0})=0.5,\;x_{3}(t_{0})=0.5,$$

we employ MATLAB 2023 simulation software in conjunction with the Milstein method to obtain numerical solutions for model (33). These results are graphically illustrated in Figure 1.

**Figure 2:**
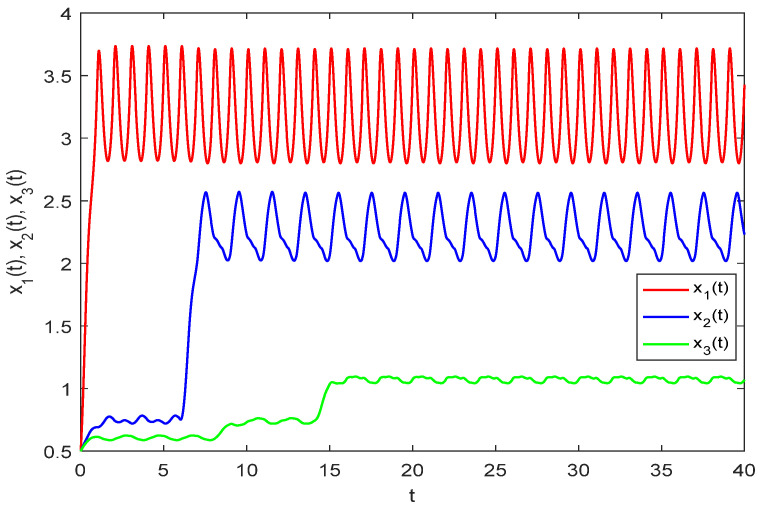
Numerical simulation of models (33)–(34) without stochastic interference.

As illustrated in Figure 1, three species starting from a given initial value exhibit random fluctuations within a defined range after a period of growth, indicating uniform persistence in model (33). Due to the time lag effect, predator and top predator populations do not increase immediately but begin to change after a buffer period. Figure 2 displays the numerical solution of model (33) without random perturbations. A comparison of Figure 1 and Figure 2 reveals that while the persistence of model (33) remains unaffected by stochastic disturbances, the solution trajectories show marked differences in their numerical values.

**Figure 3:**
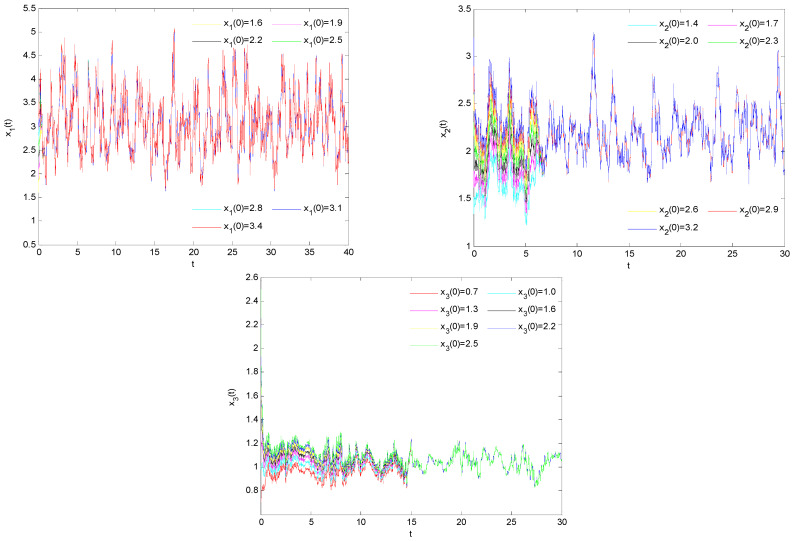
Numerical simulation of the solution of model (33) under different initial values.

To verify the global asymptotic stability of model (33), we conducted extensive numerical simulations using various positive initial conditions. The results consistently demonstrate that, regardless of the chosen initial values, model (33) exhibits asymptotic stability. As shown in Figure 3, under different initial conditions, the system’s solutions converge toward each other, gradually approaching and eventually coinciding over time. This confirms that model (33) demonstrates global asymptotic stability.

**Figure 4:**
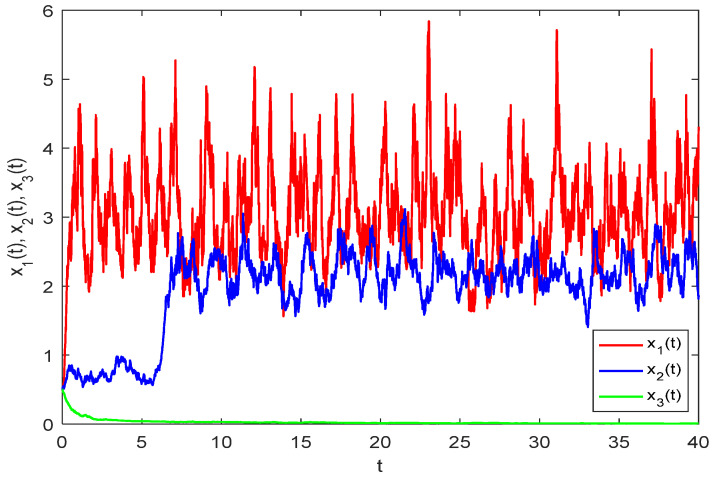
Numerical simulation of model (35) with $x_{i}(t_{0})=0.5,(i=1,2,3)$.

To further verify the validity of Theorem 2, we modify parameter $a_{32}(t)$ to $a_{32}(t)=0.001+0.0005\sin\pi t$ in model (33), thereby deriving model (35). At this parameter value, condition $\left(H_{3}\right)$ is no longer satisfied. According to the theoretical analysis, system (35) should consequently lose uniform persistence. With a time delay parameter $\tau_{1}=6,\tau_{2}=8$, a step size Δt=0.01, and initial conditions $x_{i}(t_{0})=0.5,(i=1,2,3)$, we perform numerical simulations on system (35) using the Milstein method. These results are graphically illustrated in Figure 4.

(35) $$\left\{\begin{array}{l} \begin{array}{l} dx_{1}(t)=x_{1}(t)\left[\left(5.5+0.5\sin\pi t)-(1.2+1\left|\sin\pi t\right|)x_{1}(t\right)\right.\\ \;\;\;\left.-\frac{\left(0.062+0.0005\sin\pi t)x_{2}(t\right)}{1+(0.5+0.1\sin\pi t)x_{1}(t)+(0.11+0.01\sin\pi t)x_{2}(t)}\right]dt+(0.5+0.1\left|\sin\pi t\right|)x_{1}(t)dB_{1}(t), \end{array}\\ \begin{array}{l} dx_{2}(t)=x_{2}(t)\left[-(0.005+0.001\left|\cos\pi t\right|)+\frac{\left(5.5+0.5\left|\sin\pi t\right|)x_{1}(t-\tau_{1}\right)}{1+(0.5+0.1\sin\pi t)x_{1}(t-\tau_{1})+(0.11+0.01\sin\pi t)x_{2}(t-\tau_{1})}\right.\\ \;\;\;\left.-(2.75+0.25\left|\cos\pi t\right|)x_{2}(t)-\frac{\left(0.03+0.004\sin\pi t)x_{3}(t\right)}{1+(1.5+0.2\left|\sin\pi t\right|)x_{2}(t)+(0.11+0.01\sin\pi t)x_{3}(t)}\right]dt\\ \;\;\;+(0.2+0.1\left|\sin\pi t\right|)x_{2}(t)dB_{2}(t), \end{array}\\ \begin{array}{l} dx_{3}(t)=x_{3}(t)\left[-(0.002+0.001\left|\sin\pi t\right|)+\frac{\left(0.001+0.0005\sin\pi t)x_{2}(t-\tau_{2}\right)}{1+(1.5+0.2\left|\sin\pi t\right|)x_{2}(t-\tau_{2})+(0.11+0.01\sin\pi t)x_{3}(t-\tau_{2})}\right.\\ \;\;\;-(4.25+0.2\left|\cos\pi t\right|)x_{3}(t)]dt+(0.15+0.075\sin\pi t)x_{3}(t)dB_{3}(t). \end{array} \end{array}\right.$$

As illustrated in Figure 4, following an initial increase, both the prey and predator populations exhibit random fluctuations within specific boundaries. Conversely, the top predator population demonstrates a continuous decline over time, eventually leading to extinction. These observations provide further validation for the theoretical analysis concerning the uniform persistence of model (2).

In order to further prove the correctness of Theorem 3, we give the following model

(36) $$\left\{\begin{array}{l} \begin{array}{l} dx_{1}(t)=x_{1}(t)\left[\left(3.5+1.5\sin\pi t)-(1.5+1\left|\sin\pi t\right|)x_{1}(t\right)\right.\\ \;\;\;\left.-\frac{\left(0.15+0.1\sin\pi t)x_{2}(t\right)}{1+(2+0.5\sin\pi t)x_{1}(t)+(1.2+0.5\sin\pi t)x_{2}(t)}\right]dt+(0.5+0.1\left|\sin\pi t\right|)x_{1}(t)dB_{1}(t), \end{array}\\ \begin{array}{l} dx_{2}(t)=x_{2}(t)\left[-(0.02+0.01\left|\cos\pi t\right|)+\frac{\left(4+0.5\left|\sin\pi t\right|)x_{1}(t-\tau_{1}\right)}{1+(2+0.5\sin\pi t)x_{1}(t-\tau_{1})+(1.2+0.5\sin\pi t)x_{2}(t-\tau_{1})}\right.\\ \;\;\;\left.-(1.5+0.25\left|\cos\pi t\right|)x_{2}(t)-\frac{\left(0.15+0.1\sin\pi t)x_{3}(t\right)}{1+(0.25+0.1\left|\sin\pi t\right|)x_{2}(t)+(2.5+0.5\left|\sin\pi t\right|)x_{3}(t)}\right]dt\\ \;\;\;+(0.2+0.1\left|\sin\pi t\right|)x_{2}(t)dB_{2}(t), \end{array}\\ \begin{array}{l} dx_{3}(t)=x_{3}(t)\left[-(0.0005+0.0001\left|\sin\pi t\right|)+\frac{\left(2+0.5\sin\pi t)x_{2}(t-\tau_{2}\right)}{1+(0.25+0.1\left|\sin\pi t\right|)x_{2}(t-\tau_{2})+(2.5+0.5\left|\sin\pi t\right|)x_{3}(t-\tau_{2})}\right.\\ \;\;\;\left.-(7.25+0.25\left|\cos\pi t\right|)x_{3}(t)\right]dt+(0.015+0.0075\sin\pi t)x_{3}(t)dB_{3}(t). \end{array} \end{array}\right.$$

By simple calculation, we obtain that

$$\begin{array}{l} M_{1}=3.25,\;\;M_{2}=1.99,\;\;M_{3}=1.36,\;\;m_{1}=0.61,\;\;m_{2}=0.03,\;m_{3}=0.001,\\ r_{1}^{m}-0.5{\left(\sigma_{1}^{l}\right)}^{2}=4.875,\;\;a_{21}^{m}/\beta_{1}^{l}-r_{2}^{l}-0.5{\left(\sigma_{2}^{l}\right)}^{2}=2.985,\;\;a_{32}^{m}/\beta_{2}^{l}-r_{3}^{l}-0.5{\left(\sigma_{3}^{l}\right)}^{2}=9.86,\\ r_{1}^{l}-a_{12}^{m}/\gamma_{1}^{l}-0.5(\sigma_{1}^{m})=1.525,\;\;a_{21}^{l}m_{1}/(1+\beta_{1}^{m}M_{1}+\gamma_{1}^{m}M_{2})-r_{2}^{m}-a_{23}^{m}/\gamma_{2}^{l}-0.5{\left(\sigma_{2}^{m}\right)}^{2}=0.053,\\ a_{32}^{l}m_{2}/(1+\beta_{2}^{m}M_{2}+\gamma_{2}^{m}M_{3})-r_{3}^{m}-0.5{\left(\sigma_{3}^{m}\right)}^{2}=0.0075,\;\;A=-4.09,\;\;B=-17.73,\;\;C=-7.80. \end{array}$$

**Figure 5:**
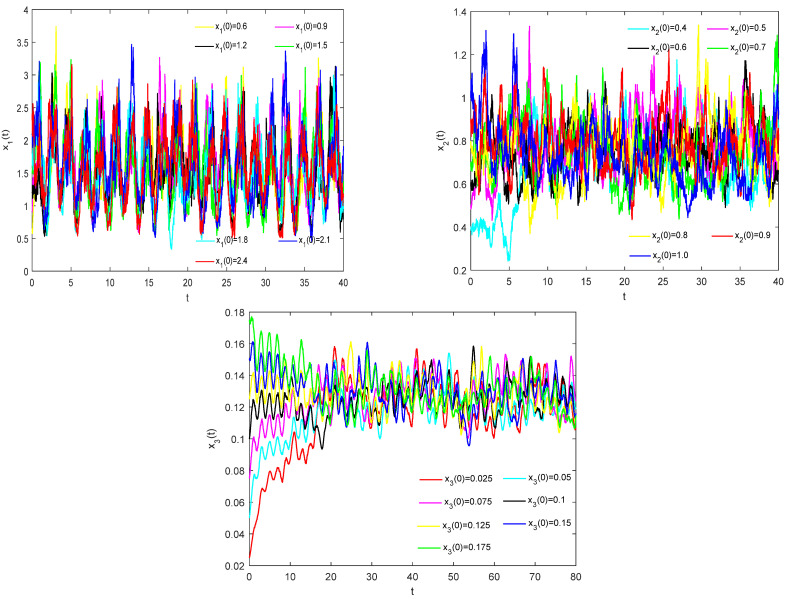
Numerical simulation of the solutions of model (36) under different initial values.

It can be seen from the above calculation that model (36) satisfies the conditions of Theorem 2 but does not satisfy those of Theorem 3. In accordance with Theorems 2 and 3, model 36 exhibits persistence yet lacks global asymptotic stability. By implementing the time delays $\tau_{1}=6,\tau_{2}=8$, step length Δt=0.01, and different initial conditions, we employ MATLAB 2023 simulation software in conjunction with the Milstein method to obtain numerical solutions for system (36). These results are graphically illustrated in Figure 5. As shown in Figure 5, under different initial conditions, the model’s solutions do not converge. This can further verify the correctness of the theoretical analysis on the stability of model (1.2).

**Remark 1.**  *The theoretical results presented in this paper hold significant biological implications that advance our understanding of complex ecological dynamics and inform conservation strategies. By developing a novel stochastic multi-species predator–prey model incorporating time delays and the Beddington–DeAngelis functional response, this research provides critical insights into the mechanisms governing species coexistence and ecosystem stability under environmental stochasticity. The biological significance of this work lies in its ability to mathematically characterize the delicate balance between persistence and extinction in ecological communities. The established uniform persistence criteria reveal how noise intensities, delay bounds, and interaction coefficients collectively determine the long-term survival of all species. This finding has profound implications for predicting ecosystem responses to environmental perturbations, particularly in the face of climate change and habitat fragmentation that introduce increasing levels of uncertainty into ecological systems.*

Numerical simulations validate these theoretical predictions, demonstrating that while moderate environmental stochasticity maintains species diversity through bounded density fluctuations, excessive noise or inappropriate time delays can destabilize the system. The observed extinction of the top predator when theoretical conditions are not met highlights the critical thresholds that must be maintained to prevent cascading biodiversity losses in real-world ecosystems. From a conservation perspective, the research provides a quantitative framework for evaluating management interventions. The demonstrated global stability suggests that maintaining interaction parameters within specific bounds could ensure ecosystem resilience against random environmental fluctuations. This insight is particularly valuable for designing protected areas and implementing harvesting quotas that account for both direct species interactions and indirect environmental effects. The integration of Beddington–DeAngelis functional responses with stochastic delay differential equations represents a methodological breakthrough in ecological modeling. By capturing realistic predator–prey dynamics that account for saturation effects and interference, the model offers improved predictive capacity compared to traditional ratio-dependent approaches.

Ultimately, this work bridges the gap between abstract mathematical theory and applied conservation biology. The rigorous stochastic framework not only enhances our fundamental understanding of ecological complexity but also provides actionable insights for maintaining biodiversity in an increasingly unpredictable world. By identifying the precise conditions under which multi-species communities maintain stability, the research contributes to the development of proactive management strategies that preserve ecological integrity amid global environmental change.

## 6. Conclusions

This paper presents a novel stochastic multi-species predator–prey model integrating time delays and the Beddington–DeAngelis functional response, significantly advancing ecological modeling under environmental stochasticity. The study establishes the existence and uniqueness of a global positive solution for any positive initial value, derives sufficient conditions for the uniform persistence of all species, and investigates the global attractivity of the system’s solutions. By constructing sophisticated Lyapunov functions tailored to the model’s stochastic and delayed characteristics, rigorous criteria are provided to ensure the long-term survival and stability of multi-species ecosystems under random environmental disturbances and historical effects. Numerical simulations using the Milstein method corroborate these analytical results, demonstrating system permanence within bounded densities, convergence under varying initial conditions, and the extinction of the top predator when theoretical conditions are not met. This work bridges theoretical ecology and stochastic analysis, offering testable predictions for complex ecosystems under environmental volatility.

While this paper makes substantial contributions to ecological modeling, several avenues for future research emerge. Firstly, the model could be extended to incorporate more complex ecological interactions, such as additional predator or prey species, or intraguild predation. Secondly, the impact of different types of environmental stochasticity, such as telegraph noise or Lévy noise, on predator–prey dynamics could be explored. Additionally, the stability and persistence of the system under varying time delays or functional responses warrant further investigation to understand their influence on ecological balance. Finally, experimental validation using empirical data from real-world ecosystems would enhance the applicability and relevance of the theoretical findings, providing more robust tools for ecosystem management and conservation.

## Figures and Tables

**Table 1:** The biological implications associated with the parameters in models (2).

Parameter	Definition	Parameter	Definition
$r_{1}(t)$	Intrinsic growth rate	$a_{ii}(t)\;(i=1,2,3)$	Intrapatch restriction coefficients
$r_{2}(t),\;r_{3}(t)$	Death rates	$\tau_{1},\;\tau_{2}$	Delays due to pregnancy
$a_{12}(t),\;a_{23}(t)$	Predation rates	$a_{21}(t),\;a_{32}(t)$	Energy conversion rates
$\beta_{1}(t),\;\beta_{2}(t)$	Times for prey to be digested	$\gamma_{1}(t),\;\gamma_{2}(t)$	Interference coefficient among predators

## Data Availability

Data are contained within the article.
